# Effectiveness and Experiences of Self‐Management Interventions in Adults With Multiple Long‐Term Conditions: A Mixed Methods Systematic Review

**DOI:** 10.1111/jep.70565

**Published:** 2026-08-20

**Authors:** Ellen Hopwood, Michelle Hadjiconstantinou, Ashkon Ardavani, Ash Routen, Shavez Jeffers, Priscilla Katapa, Winifred Ekezie, Patrick Highton, Kamlesh Khunti, Ffion Curtis

**Affiliations:** ^1^ National Institute for Health and Care Research Applied Research Collaboration East Midlands University of Leicester Leicester UK; ^2^ Department of Population Health Sciences University of Leicester Leicester UK; ^3^ Diabetes Research Centre, College of Life Sciences University of Leicester UK; ^4^ National Institute for Health and Care Research Biomedical Research Centre University of Leicester Leicester UK; ^5^ School of Social Sciences and Humanities Aston University Birmingham UK; ^6^ Liverpool Reviews and Implementation Group (LRiG) University of Liverpool Liverpool UK

**Keywords:** chronic disease, multimorbidity, primary care, self‐management, systematic review

## Abstract

**Rationale:**

Individuals who live with multiple long‐term conditions (MLTCs) are more likely to experience poor quality of life and premature mortality. Supporting self‐management is important in reducing the health burden experienced by this population. However, evidence remains limited regarding both the effect and lived experiences of self‐management support interventions specifically designed for individuals with MLTCs.

**Aim:**

This mixed‐methods systematic review aimed to identify and summarise findings on the effectiveness and experiences of self‐management support interventions for adults living with MLTCs.

**Methods:**

MEDLINE, PsycINFO, CINAHL, Web of Science and Cochrane Library were searched from inception to July 2025. Eligible studies included adults aged 18 years or above with two or more long‐term conditions. Quantitative, qualitative and mixed‐method designs were included. Quantitative and qualitative findings were synthesised separately, with meta‐integration used to integrate quantitative and qualitative evidence where appropriate. Study quality was assessed using validated appraisal tools appropriate to each study design.

**Results:**

Twenty‐eight studies met the inclusion criteria. Quantitative evidence indicated modest improvements in specific self‐management behaviours (health‐directed behaviours, self‐monitoring and skill acquisition). However, there was little to no effect of self‐management support interventions for MLTCs on quality of life or psychological outcomes. In contrast, qualitative findings indicated perceived improvements in quality of life, mental health and self‐management behaviours, and highlighted the influence of social factors, such as relationships and finances, on engagement in self‐management interventions.

**Conclusions:**

Although modest improvements were observed in specific self‐management behaviours, there is little to no quantitative evidence that self‐management interventions improve most outcomes for people with MLTCs, including quality of life and mental health. These effects remain uncertain due to mixed findings across qualitative and quantitative studies, as qualitative findings indicated benefits for quality of life and mental health. This emphasises the need for validated, MLTC‐specific outcome measures in future research.

## 1 Introduction

Multiple long‐term conditions (MLTCs or multimorbidity) are defined as “the co‐existence of two or more long‐term conditions in a single individual” [1]. Globally, the prevalence of people with two or more long‐term conditions ranges from 19.1% to 42.4% [2, 3, 4, 5]. The number of adults with MLTCs in the United Kingdom is also expected to increase by more than half by 2035 [6]. People living with MLTCs often experience diminished quality of life, poorer mental health and premature mortality [7, 8]. MLTCs are also associated with increased visits to health services and greater health and social costs [9, 10].

Supporting self‐management of long‐term conditions is associated with improved health outcomes across a range of health conditions [11, 12]. Self‐management can involve three main tasks [13]: (i) medical management (e.g., attending healthcare appointments, collaborating with healthcare services, taking medications); (ii) behaviour management (e.g., monitoring symptoms, changing lifestyle behaviours); (iii) emotional management (e.g., managing frustrations, managing mental health). Effective self‐management reduces the burden of symptoms for individuals living with MLTCs, and can prevent the development of further complications and improve quality of life [11].

Self‐management is not an isolated responsibility and individuals living with MLTCs often require support from healthcare professionals (HCPs), family, friends and other people living with long‐term conditions [14]. Support for self‐management can encourage and empower people to manage their conditions effectively [15, 16], but it is often difficult to implement due to a complex, fragmented health system and increased symptom burden experienced by people living with MLTCs [17, 18]. Structured self‐management support can be delivered using a variety of methods [19] and by a range of providers, including HCPs [20], community health workers or peers who are also living with similar long‐term conditions [21]. Self‐management support for MLTCs can involve [14]: (i) instrumental support such as provision of knowledge on conditions or support for medical management); (ii) psychosocial support for managing the emotions associated with living with MLTCs; (iii) relational support which involves interactions and socialisation with others, including HCPs and peers.

Previous systematic reviews have shown that self‐management support interventions which target single conditions or specific comorbid conditions are effective [22, 23]. Improvements were observed across clinical outcomes in studies which targeted diabetes, chronic obstructive pulmonary disease and depression. However, interventions which target defined conditions, comorbidities or disease clusters are often more specific to these conditions and use disease‐specific outcomes. Interventions which target MLTCs often require an approach focused on less disease‐specific issues, such as physical activity, nutrition and stress management, with a person‐centred approach and self‐management support being identified as important components [24].

A previous systematic review focused on complex interventions targeting MLTCs showed little to no effect on quality of life and mental health outcomes [24]. However, this review did not include qualitative studies and there was a lack of focus on self‐management support interventions for MLTCs. Therefore, the aim of this systematic review was to summarise quantitative and qualitative findings on the effectiveness and experiences of self‐management support interventions in people with MLTCs, carers and HCPs.

## 2 Methods

A convergent segregated systematic mixed studies approach was used for this review, based on established guidance [25, 26, 27]. This approach allowed for quantitative data and qualitative data to be analysed separately, and integrated where appropriate to ascertain whether quantitative and qualitative findings supported; contradicted; or added to each other [25]. This approach offered a more comprehensive synthesis of the evidence on self‐management interventions for MLTCs, compared with a single method review [28]. Methods of analysis and eligibility criteria were specified in advance and documented in a protocol (CRD42022302959) registered on PROSPERO [29]. Ethical approval was not required for this study.

### 2.1 Eligibility Criteria

Studies were included if participants were adults aged 18 years or above with MLTCs, relatives or carers of people with MLTCs, or HCPs. MLTCs were defined as two or more long‐term health conditions [30]. Self‐management interventions targeting MLTCs were included. Eligible interventions were those addressing one or more of the eight self‑management approaches outlined by Barlow et al. [12], including information, drug and symptom management, addressing psychological consequences, lifestyle changes, social support, communication and other strategies (including goal setting, action planning and accessing support services). Interventions based in any setting were eligible. Primary outcomes included quality of life, mental health and experiences of self‐management interventions. Additional outcomes included clinical, psychological, behavioural and health service outcomes.

A range of empirical quantitative study designs were eligible. To assess intervention effects, interventional studies including randomised controlled trials (RCTs), cluster RCTs, and pre‐post interventional studies were included. Observational studies, including cohort and cross‐sectional studies, were also eligible. Including a range of quantitative designs, including uncontrolled and cross‐sectional studies, ensured comprehensive coverage of self‐management support interventions for MLTCs which is an emerging field. Limiting inclusion to RCTs could have potentially excluded relevant evidence related self‐management interventions which were developed and evaluated in real‐world settings, rather than through controlled trials. In addition, qualitative study designs, using interviews, focus groups or observational methods were included. Mixed‐method study designs were eligible if their quantitative and qualitative components could be extracted and synthesised separately.

There were no date or region restrictions. Studies focused on single index or specific comorbid conditions were excluded, as the focus of these intervention did not fit with the broader definition of MLTCs used for this review. The following were excluded: (i) studies with an intervention targeting a single index condition even if individuals had co‐occurring health conditions; (ii) studies with an intervention targeting specifically stated comorbid conditions; (iii) study populations aged under 18 years; (iv) studies not in English.

### 2.2 Information Sources and Search Strategy

The following databases were searched from inception to January 2024: MEDLINE, CINAHL, PsycINFO, Cochrane Central Register of Controlled Trials and Web of Science. The searches were updated in July 2025.

The search strategy was developed using guidance from academic librarians at the University of Leicester. The search strategy included terms related to ‘multiple long‐term conditions’, ‘multimorbidity’ and ‘self‐management’ and was adapted for use across all the databases (Figure S1). Supplementary searches were conducted with Google Scholar, ClinicalTrials.gov, and reference screening and citation tracking from included studies and relevant systematic reviews [24].

### 2.3 Study Selection

References retrieved from the database searches were stored in Endnote 20 and imported into Covidence software. Duplicate studies were removed prior to screening. Two reviewers independently screened titles and abstracts for inclusion. Subsequently, full texts were retrieved for potentially eligible studies and independently screened by two reviewers against the pre‐specified inclusion criteria. Any conflicts regarding eligibility were resolved through discussion between two reviewers or by a third reviewer.

### 2.4 Data Extraction

Data were extracted by one reviewer and checked for accuracy by a second using an adapted pre‐piloted Cochrane data extraction template. Data extracted included the characteristics of the studies, populations and interventions, and the main results for the primary and secondary outcomes.

### 2.5 Quality Appraisal

Quality assessment of the included studies was performed independently by two reviewers. Given the different study designs included in this systematic review, multiple quality appraisal tools were utilised to ensure that each study was rigorously assessed, using a checklist tailored to the methodological approach of each study.

The Critical Appraisal Skills Programme (CASP) checklist, which is a widely accepted quality appraisal tool in systematic review practice, was used for RCTs and qualitative studies [31]. The National Heart, Lung and Blood Institute (NHLBI) quality assessment tool [32] was used for the before‐after studies and for the cross‐sectional studies, as these checklists were specifically designed for these study types. The Mixed Methods Appraisal Tool (MMAT) was used to assess mixed methods studies, which specifically assesses quality of mixed‐methods designs [33].

To determine the overall quality rating (good, unclear or poor) for each study, tool‐specific criteria were applied independently by two reviewers. After independently appraising each study using the appropriate checklist, the results were reviewed collaboratively by the two reviewers. Each study was discussed in detail, with consideration on how many criteria were met and the relative importance of met and unmet items. Agreement was met on overall quality ratings that reflected both the checklist scores and reviewer judgement. Discrepancies between the two reviewers were resolved through further discussion.

### 2.6 Data Synthesis

Study characteristics were summarised in tables based on study type. A narrative synthesis was created from the included studies structured around outcome measures, with an effect direction plot used to present findings for each outcome, using symbols to indicate positive, negative or similar results [34, 35].

Where quantitative studies overlapped in terms of outcome measures, data were combined using a random effects meta‐analysis in RevMan Web. Authors of the primary studies were contacted to request additional data for the meta‐analysis. Meta‐analysis calculated a single pooled estimate and 95% confidence intervals (95% CI). Mean differences (MD) were used where outcomes were assessed using the same measurement tool and standardised mean differences (SMD) were applied when an outcome was measured using different tools. Heterogeneity was quantified using the *I* ^2^ statistic, with a threshold of > 40% indicating moderate to substantial heterogeneity [36]. A sensitivity analysis was performed when heterogeneity was > 40%, using a ‘one‐study removed’ approach to establish the effect that removing one study at a time had on between‐study heterogeneity [37].

The qualitative analysis was conducted in accordance with the Thomas and Harden thematic synthesis approach [38]. The first stage involved generating initial codes from the studies using an inductive approach. The second stage was the development of descriptive themes from these codes. Stage three involved using the aim of this review as a framework to generate additional concepts and produce final analytical themes.

The quantitative and qualitative results were analysed separately, and then integrated where appropriate. Integration involved a three‐stage comparative process: (i) identifying areas of overlap across quantitative outcome domains and qualitative themes; (ii) examining whether qualitative findings complemented, expanded or refuted the quantitative results; (iii) producing an interpretive statement for each outcome category [39]. Quantitative findings served as the initial point of reference and qualitative findings were used to provide explanation, context or contrast.

## 3 Results

**Figure 1:**
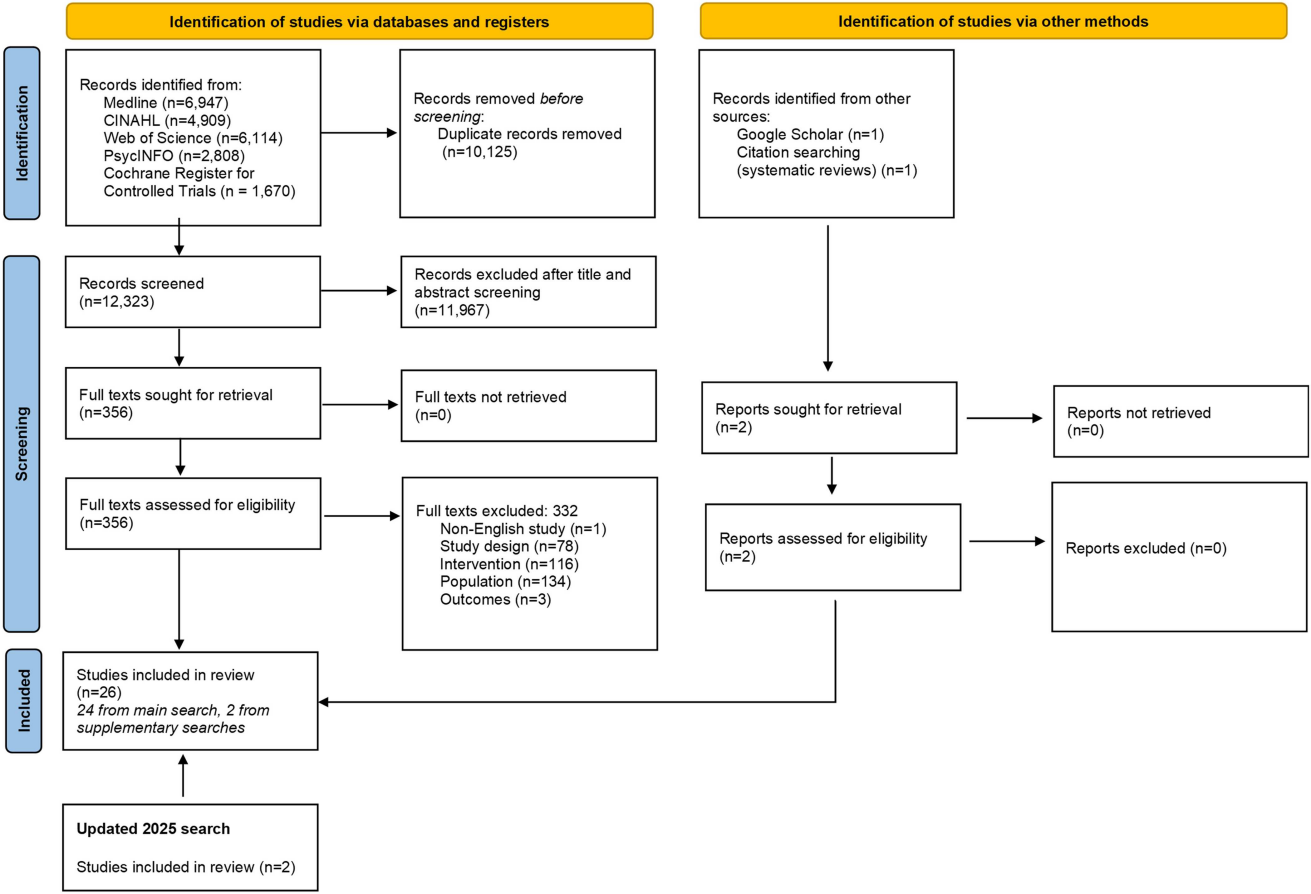
Preferred Reporting Items For Systematic Reviews and Meta‐Analyses (PRISMA) flow chart of the literature database search. Flow chart showing the number of studies identified via databases, registers and other methods, and the numbers excluded at each stage of the systematic review process.

A total of 12,323 references excluding duplicates were identified. Of these, 24 studies met the eligibility criteria (Figure 1). An additional two studies were identified through supplementary searches, resulting in a total of 26 studies included in the review. The updated 2025 search brought this total up to 28 studies.

### 3.1 Study Designs

Eighteen quantitative studies (Table 1), five qualitative studies (Table 2) and five mixed‐methods studies were identified (Table 3). Quantitative studies consisted of 15 RCTs, two before‐after studies and one cross‐sectional study. The studies were published between 2000 and 2025. The included studies were conducted in Australia (*n* = 1), Canada (*n* = 8), China (*n* = 1), Denmark (*n* = 2), Ireland (*n* = 4), South Korea (*n* = 2), Switzerland (*n* = 1), the UK (*n* = 4) and the United States of America (*n* = 4). One study was conducted across Ireland and Belgium simultaneously.

**Table 1:** Quantitative studies. Summary of the characteristics of quantitative studies based on study design, population, intervention, outcomes and study findings.

References	Study design country	Population	Intervention	Comparator	Primary and secondary outcome(s)	Key findings
Boult et al. [40]	Cluster RCT United States of America	Multimorbid, high risk of health service use, aged 65 or over Total: *n* = 904 Intervention: *n* = 485 Control: *n* = 419 Males: *n* = 383 Females: *n* = 467 Ethnicity: white *n* = 435 Age: 77.1 (int); 77.8 (con) Conditions: 4.3 (int); 4.3 (con)	Components: assessment and monitoring, action plan development, coordinate transitions of care, educating and promoting self‐management, motivational interviewing, facilitating access to resources Setting: community based, in patients' homes Duration: 20 months, monthly monitoring by nurses Delivery: led by HCPs; in‐person; individually Follow‐up: baseline, 8 months, 20 months	Usual care (from established primary care physicians)	Health service use	Reduced home healthcare episodes by 30% compared with usual care. Little effect on the use of other health services, including hospital admissions, hospital days, readmissions, emergency department visits, primary care visits and specialist visits.
Contant et al. [41]	RCT Canada	Three or more conditions, aged 18 to 75 Total: *n* = 281 Intervention: n = 139 Control: *n* = 142 Males: *n* = 72 (con); *n* = 69 (int) Females: *n* = 70 (con); *n* = 70 (int) Age: 53.3 ± 11.1 (int); 53.7 ± 11.3 (con) Conditions: 5.7 (int); 5.3 (con)	Components: self‐management support with a health education and a person‐centred motivational approach, involving an individualised care plan Setting: primary care Duration: 3 months, with at least three encounters Delivery: led by HCPs; in‐person; individually Follow‐up: baseline and 3 months	Usual care (received intervention after 3 months waiting period)	Self‐management capacity and skills	Statistically significant improvements across six of the eight domains for self‐management: skill and technique acquisition, health directed behaviour, emotional well‐being, self‐monitoring and insight, constructive attitudes and approaches, health service navigation, indicating a moderate positive effect of the intervention.
Fortin et al. [42]	Before‐after Canada	Three or more chronic conditions, aged 18 to 80 Total: *n* = 284 Males: *n* = 132 Females: *n* = 152 Age: 60.9 ± 10.4 Conditions: 5.0	*Same intervention as Fortin2021* [64] Components: patient‐centred care, self‐management support, interprofessional collaboration and motivational interviewing, involving an individualised care plan developed in accordance with patients' goals Setting: primary care Duration: 4 months, with visits dependent on patient needs Delivery: led by HCPs; in‐person; individually Follow‐up: baseline and 12 months	No comparator	Primary outcomes: self‐management; self‐efficacy; health status; quality of life; psychological distress; health behaviours	Statistically significant improvements in emotional well‐being, physical and mental quality of life, psychological distress and healthy eating compared with baseline. Subgroup analysis showed that younger people, people with a lower number of conditions and higher income may show more positive outcomes related to self‐management, health status and health behaviours.
Garvey et al. [43]	RCT Ireland	Two or more chronic conditions, minimum of four repeat medications, aged over 18 Total: *n* = 50 Intervention: *n* = 26 Control: *n* = 24 Males: *n* = 9 (con); *n* = 9 (int) Females: *n* = 15 (con); *n* = 17 (int) Age: 65 (int) 67.5 (con) Conditions: 4.0 (int); 5.0 (con)	Components: included occupational therapy, peer support in group meetings and goal setting based on prioritisation of patient preferences; focused on self‐management, fatigue and energy management, managing mental health, physical activity, healthy eating, managing medications, communication and goal setting. Setting: primary care Duration: 6 weeks, with a session held weekly Delivery: led by occupational therapists, with peer support incorporated; in‐person; group Follow‐up: baseline and 2 weeks after intervention	Usual care (invited to intervention once trial is completed)	Primary outcomes: frequency of activity participation Secondary outcomes: self‐perception of satisfaction with and ability to perform daily activities; independence in activities of daily living; anxiety and depression; self‐efficacy; health‐related quality of life; self‐management; healthcare use; individualised goal attainment	Significant improvement in the frequency of activity participation compared with the control group. Improvements in self‐perception of satisfaction and ability to perform daily activities, independence in activities of daily living, self‐efficacy and EQ‐VAS component of quality of life. No differences were observed in self‐management, healthcare utilisation and the EQ‐5D domain of quality of life.
Hwang et al. [44]	RCT South Korea	Older adults aged 65 to 80 years, living with two or more chronic conditions, taking more than one medication Total: *n* = 42 Intervention: *n* = 21 Control: *n* = 21 Males: *n* = 7 (con); *n* = 3 (int) Females: *n* = 14 (con); *n* = 18 (int) Age: 65‐74 = 14; 75‐84 = 7 (int) 65‐74 = 17; 75‐84 = 4 (con) Conditions: 3.95 ± 1.53 (int); 5.0 ± 2.08 (con)	Components: digital health coaching self‐management programme, consisting of education using videos, quizzes and in person group session; individual health coaching and self‐monitoring Setting: hospital and home Duration 8 weeks, with weekly telephone health coaching appointments Delivery: led by HCPs, with self‐learning encouraged; in‐person and remote, with individual and group sessions Follow‐up: baseline and immediately upon completion of intervention	Usual care (received education session and educational video clips once trial completed)	Primary outcomes: self‐management behaviours; medication adherence Secondary outcomes: health status; quality of life	Medication adherence improved significantly in the intervention group compared with control group. However, no differences were observed in self‐management behaviours. Health status measures, including disease‐related distress and depression, were significantly improved in the intervention group. However, other measures such as symptoms of disease (fatigue, sleep, pain, stress and shortness of breath), and self‐rated health showed no significant differences. No significant changes were observed in quality of life.
Khunti et al. [45]	RCT United Kingdom	Two or more chronic conditions, aged 40 to 85 Total: *n* = 353 Intervention: *n* = 180 Control: *n* = 173 Males: *n* = 80 (con); *n* = 81 (int) Females: *n* = 93 (con); *n* = 99 (int) Age: 67.03 ± 9.82 (int) 68.52 ± 8.85 (con) Conditions: 4.46 (int); 4.17 (con) Ethnicity: White 97.11%; South Asian 2.31%; Other 0.58% (con); White 96.11%; South Asian 2.83%; Other 0.57% (int)	Components: person‐centred self‐monitoring and goal setting, focused on increasing physical activity and supporting self‐management challenges; supported by provision of resistance bands and regular reminder text messages Setting: primary care and community Duration: 8 weeks for group sessions, with one session every 2 weeks; text messages continued for 12 months Delivery: led by professionals; in‐person and group delivery with individual text messages Follow‐up: baseline, 6 months and 12 months	Usual care (usual disease management as required and received intervention once trial completed)	Primary outcomes: change in volume of daily physical activity Secondary outcomes: physical activity related secondary outcomes; clinical venous blood and anthropometric measures; quality of life; anxiety and depression; self‐efficacy; dietary behaviours	Led to a small reduction in daily physical activity levels in the intervention group and control group, with a greater reduction in the intervention group. No statistically significant differences between groups in any of the secondary outcomes.
Lear et al. [46]	RCT Canada	Two or more chronic conditions, aged 19 or over Total: *n* = 230 Intervention: n = 117 Control: *n* = 113 Males: *n* = 69 (con); *n* = 72 (int) Females: *n* = 44 (con); *n* = 44 (int) Age: 69.6 ± 8.8 (int) 71.3 ± 9.5 (con) Conditions: 2	Components: internet based self‐management programme with support via telephone nurses and integration within primary care; involved participants setting their own targets Setting: primary care and internet‐based Duration: 2 years Delivery: led by HCPs using internet platform; digitally; individually Follow‐up: baseline, 12 months and 24 months	Usual care (given educational information regarding chronic disease management and a list of internet‐based resources)	Primary outcome: all cause hospitalisations Secondary outcomes: hospital length of stay; quality of life; self‐management; social support; user login data.	No significant reduction in hospitalisations, but 25 fewer hospitalisations and 229 fewer in‐hospital days occurred in the intervention group. Four domains of self‐management and two domains of social support significantly improved in the intervention group compared with control group.
Mercer et al. [47]	Cluster RCT United Kingdom	Two or more long‐term conditions, aged 30 to 65 Total: *n* = 152 Intervention: n = 76 Control: *n* = 76 Males: *n* = 37 (con); *n* = 30 (int) Females: *n* = 39 (con); *n* = 46 (int) Age: 51.9 ± 9.6 (int) 53.1 ± 8.0 (con) Conditions: 4.8 (int); 5.1 (con)	Components: whole‐system primary care intervention to allow longer consultations with patients to identify patient priorities, support self‐management and generate a care plan Setting: primary care Duration: 12 months with 3 consultations on average per patient Delivery: led by HCPs; in‐person; individually Follow‐up: baseline, 6 months and 12 months	Usual care (treatment as usual)	Primary outcomes: health related quality of life; well‐being Secondary outcomes: anxiety and depression; self‐efficacy; self‐esteem; level of engagement and retention of practices and patients in the trial	Improved health related quality of life at 6 months but this was not maintained at 12 months. There was a significant reduction in negative wellbeing at 12 months, but positive well‐being, energy and general wellbeing was not significantly changed by the intervention. None of the secondary outcomes were significantly changed by the intervention.
O'Toole et al. [48]	RCT Ireland	Two or more chronic conditions; 4 or more repeat medications; aged 40 or over Total: *n* = 149 Intervention: *n* = 78 Control: *n* = 71 Males: *n* = 21 (con); *n* = 25 (int) Females: *n* = 50 (con); *n* = 53 (int) Age: 65.5 ± 9.3 (int) 65.9 ± 10.5 (con) Conditions: 4.4 (int); 4.7 (con)	*Same intervention as Garvey2015* [43] Components: included occupational therapy, peer support in group meetings and goal setting based on prioritisation of patient preferences; focused on self‐management, fatigue and energy management, managing mental health, physical activity, healthy eating, managing medications, communication and goal setting. Setting: primary care and community centres Duration: 6 weeks, with a session held weekly Delivery: led by occupational therapists; in‐person; group Follow‐up: baseline, immediately post‐intervention and 6 months	Usual care (on wait list for intervention)	Primary outcomes: health related quality of life; frequency of activity participation Secondary outcomes: independence in daily life; self‐efficacy; anxiety and depression; occupational performance; healthcare use	Improvements in health‐related quality of life immediately post intervention, but this was not maintained at 6 months. There was no change in frequency of activity participation at any time point. Perception of occupational satisfaction and number of hospital outpatient appointments were improved significantly, with no other changes across the rest of the secondary outcomes.
Panagioti et al. [49]	RCT (within a cohort) United Kingdom	Two or more long‐term conditions, aged 65 or over Total: *n* = 1306 Intervention: *n* = 504 Control: n = 802 Males: *n* = 357 (con); *n* = 232 (int) Females: *n* = 441 (con); *n* = 270 (int) Age: 75.4 ± 6.8 (int) 74.2 ± 6.4 (con) Ethnicity: white *n* = 1275; non‐white n = 23 Conditions: 6.8 (int); 6.8 (con)	Components: health coaching intervention focused on self‐management and tailored to patient, involved promoting healthy behaviours through motivation, social prescribing involving links to resources in wider community and low intensity support for low mood Setting: community based Duration: 6 months, with a phone call monthly Delivery: led by HCPs supported by specialist nurse and research team; remote (by telephone); individually Follow‐up: baseline, 6 months, 12 months and 20 months	Usual care (usual NHS care)	Primary outcomes: self‐management; quality of life Secondary outcomes: depression; self‐care; number of days they engage in health and unhealthy behaviours	This health coaching intervention did not lead to significant improvement in any of the patient reported outcomes
Park et al. [50]	RCT South Korea	Diagnosis of two or more chronic diseases within 1 year prior to the study; residents of a nursing home; aged 65 or over Total: *n* = 43 Intervention: *n* = 22 Control: *n* = 21 Males: *n* = 6 (con); *n* = 3 (int) Females: *n* = 15 (con); *n* = 19 (int) Age: 77.3 ± 7.0 (int) 78.0 ± 6.2 (con)	Components: health coaching self‐management intervention; involved individualised goal setting and support for self‐management, group education for self‐management and exercise and support for participants to achieve goals. Setting: nursing homes Duration: 8 weeks, with a group and individual session weekly Delivery: led by HCPs (trained geriatric nurses); in‐person; individually and group Follow‐up: baseline and 8 weeks	Usual care (maintain lifestyle, dietary, exercise habits)	Primary outcomes: self‐management behaviours, self‐efficacy and health status Secondary outcomes: health goal setting and attainment scales	This intervention significantly improved all outcomes for self‐management behaviours, except for communication with health professionals. Self‐efficacy and self‐rated health status were also significantly improved in the intervention group compared with control.
Pellet et al. [51]	Before‐after Switzerland	Two or more chronic conditions, aged 50 years or over, hospitalised for more than 48 h Total: *n* = 138 Intervention: n = 70 Control: *n* = 68 Males: *n* = 40 (con); *n* = 35 (int) Females: *n* = 28 (con); *n* = 35 (int) Age: 75.7 ± 10.6 (con); 73.5 ± 9.8 (int) Conditions: 4.4 (con); 4.1 (int)	Components: intervention to assess patient priorities related to the transition from hospital to home and current activation for self‐management, using a teaching guide adapted to patient needs and priorities. This focused on reason for hospitalisation, warning signs, medication plan, health behaviours, following appointments and which person to contact when needed. Setting: secondary care (hospital) Duration: from admission to discharge Delivery: led by nurses; in person; individually Follow‐up: day of discharge and 7‐10 days post discharge	Control group received usual discharge preparation, focused on care coordination and less on teaching content	Patient activation measures; readiness for hospital discharge; experiences with care; readmission	Patient activation for self‐management was significantly improved 7 to 10 days post‐discharge. There were no differences between groups in readiness for discharge, discharge care experiences or readmission rates.
Reed et al. [52]	RCT Australia	Two or more chronic diseases, aged 60 years or over Total: *n* = 254 Intervention: *n* = 127 Control: *n* = 127 Males: *n* = 50 (con); *n* = 52 (int) Females: *n* = 77 (con); *n* = 75 (int) Age: 60‐75 – 61; 76‐85 – 46; > 85 − 2 (int) 60‐75 – 58; 76‐85 – 51; > 85 − 18 (con) Conditions: 4.4 (int); 4.5 (con)	Components: self‐management support programme based on cognitive behavioural therapy and motivational interviewing; involved development of individualised care plans to address key self‐management issues Setting: primary care and home visits Duration: 6 months with 3 home visits and four telephone calls Delivery: led by HCPs; individually, in‐person and remotely (via telephone) Follow‐up: baseline and 6 months	Control group: received health information relevant to conditions, non‐directive counselling, supportive listening and scheduled contact with clinicians, no self‐management tools provided	Primary outcomes: self‐rated health Secondary outcomes: health status; health behaviours; self‐efficacy; self‐management; healthcare use	Participants in the intervention group were more likely to report improvement in self‐rated health compared with control group. There were no changes reported for the secondary outcomes.
Skou et al. [53]	RCT Denmark	Adults aged 18 or over, with two or more chronic conditions Total: *n* = 228 Intervention: *n* = 115 Control: *n* = 113 Males: *n* = 60 (con); *n* = 69 (int) Females: *n* = 53 (con); *n* = 45 (int) Age: 70.0 ± 8.7 (int) 69.6 ± 8.1 (con) Conditions: 7 ± 2.9 (int); 7 ± 2.8 (con)	Components: personalised, supervised exercise therapy and self‐management support programme, using supervised exercise sessions and self‐management support sessions aimed to improve self‐management skills and motivation to maintain an active lifestyle. Setting: home based and department at hospital Duration: 24 weeks, with twice weekly sessions Delivery: led by physiotherapists and healthcare professionals, with individual and group sessions in person Follow‐up: 4 months and 12 months	Usual care	Primary outcome: quality of life Secondary outcomes: functional performance; illness burden; depression; anxiety; self‐efficacy	There was a statistically significant improvement in quality of life between the intervention and usual care groups. Although the results from the secondary outcomes favoured the intervention group, no statistically significant differences were observed between baseline and 12 months.
Sommers et al. [54]	Concurrent controlled cohort study United States of America	Undergoing treatment for two or more chronic conditions, aged 65 or over Total: *n* = 543 Intervention: *n* = 280 Control: *n* = 263 Males: 87 (con); 84 (int) Females: 176 (con); 196 (int) Age: 78.0 ± 6.8 (int) 77.0 ± 6.6 (con) Ethnicity: 80% white (con); 84% white (int)	Components: assessment of the patient and development of a risk reduction plan with set objectives and recommended self‐management strategies; monitored throughout to check disease status, any problems, coach self‐management skills and promote use of community services Setting: primary care and meetings in homes Duration: average 14 months (ranged from 10 to 18 months), monitored once every 6 weeks Delivery: led by HCPs; updates via in person visits, phone or group sessions Follow‐up: baseline, 1 year and 2 years	Usual care (from primary care physician)	Changes in number of hospital admissions, readmissions, office visits, emergency department visits, skilled nursing facility admissions, home care visits; changes in patient self‐rated physical, emotional and social functioning	The intervention significantly reduced hospital admissions in the intervention group compared with the control group. No differences were seen in health status 1 year into the intervention. Two years into the intervention, a higher number of social activities were reported by intervention participants and fewer symptoms and overall health improved.
Struwe et al. [55]	RCT (secondary analysis) United States of America	Patients with multimorbidity who had completed 75% or more of the self‐management intervention Total: *n* = 91 Intervention: *n* = 47 Control: *n* = 44 Males: 43 Females: 48 Age: 64.25 ± 14.36 Ethnicity: 93% Caucasian; 4% Native American; 2% African American: 1% Hispanic	Components: promoted patient activation by helping patients understand the importance of self‐management via education, goal setting and encouragement Setting; primary care Duration: lasted 4‐8 weeks depending on group Delivery: led by HCPs; delivered both in‐person and remotely; individually Follow‐up: baseline, completion of intervention (4 or 8 weeks), 6 months	Usual care	Patient activation measures; healthcare use; quality of life	Intervention groups showed greater improvement in patient activation compared with usual care, however many individuals in these groups did not increase the patient activation measure by 5 or more points. Those who increased the patient activation measure by 5 or more points had higher healthcare utilisation. Those who had less than 5‐point patient activation measure change showed a small, negative decrease in quality of life.
Williams‐Livingston et al. [56]	Cross‐sectional survey United States of America	High risk patients with multiple morbidities, aged 40 to 75 years Total: *n* = 93 Males: 24 Females: 69 Age: 59	Components: consisted of home and clinic visits with community health workers and their primary care physician to develop an individualized care plan to encourage and support self‐care management, provide link to community supports and community engagement activities Setting: community and primary care Duration: 90 days Delivery: led by HCPs and community health workers; in‐person; individually Follow‐up: completion of intervention	No control group	Assessment of community support utilisation; self‐care management; assessment of linkages to community support	This intervention was successful at linking participants with community support (e.g., transport, employment assistance, smoking cessation) and participants were satisfied with the benefits of these supports. There was a culture shift in practice of family medicine with a focus on community as well as the individual.
Yang et al. [57]	RCT China	At least 3 conditions; at least one medication prescribed for a condition over at least 3 months, aged 60 or over Total: *n* = 136 Intervention: *n* = 67 Control: *n* = 69 Males: 33 (con); 21 (con) Females: 36 (con); 46 (int) Age: 70.76 ± 7.49 (int) 72.67 ± 7.64 (con) Conditions: 4.73	Components: self‐management programme to improve medication adherence, medication self‐management capacity and treatment experiences. Setting: primary care and community health centres Duration: lasted 6 weeks, with 3 face‐to‐face sessions (over 4 week period) and 2 weekly follow up phone calls Delivery: led by HCPs; in‐person and remotely (phone calls); individually Follow‐up: baseline, immediately after completion, 3 months	Usual care (usual medication management and support by community general medical practitioners)	Primary outcomes: medication adherence Secondary outcomes: medication knowledge; medication beliefs; medication social support; medication self‐efficacy; medication skills; medication treatment satisfaction; treatment burden; quality of life; healthcare service use	This intervention significantly improved medication adherence immediately post‐ intervention but this was not maintained at 6 months. Knowledge and medication self‐efficacy were significantly improved immediately post‐intervention and 6 months. No changes were seen in quality of life, self‐rated health and healthcare services utilisation.

Abbreviations: con = control group, EQ‐5D = EuroQol‐5D, EQ‐VAS = EuroQol visual analogue scale, GPs = general practitioners, HCPs = heath care professionals, int = intervention group, MLTCs = multiple long‐term conditions, N/A = not applicable, NHS = National Health Service, RCT = randomised controlled trial.

**Table 2:** Qualitative studies. Summary of the characteristics of qualitative studies based on study design, population, intervention, outcomes and study findings.

References	Study design country	Population	Intervention	Comparator	Primary and secondary outcome(s)	Key findings
Doyle et al. [58]	Qualitative (interviews and focus groups) Ireland and Belgium	Two or more conditions, aged 65 years or over Triage nurses Patients: *n* = 120 Gender: Ireland: Males: *n* = 36; females: *n* = 24; Belgium: Males: *n* = 42; females *n* = 17 Age: Ireland 74.23 ± 6.4 (65‐92); Belgium 73.88 ± 6.23 (65‐91) Professionals: *n* = 7	Components: digital health platform to support self‐management by using sensing devices to measure symptoms and wellbeing, provision of educational resources related to conditions, goal setting related to physical activity and allows the user to add people to care network. Nurses are able to view and respond to alerts from the platform. Setting: digital based platform Duration: 12 months with monthly check in calls with nurses Delivery: led by nurses; remotely; individually Follow‐up: completion of intervention	N/A	Experiences of using the digital platform	Provision of patient data allowed nurses to view the whole person and not focused on a single condition or issue. Nurses provided education and motivation to users of the digital platform and appreciated being able to spend more time with patients to discuss issues and provide person‐centred care. Patients appreciated the support of the nurses and benefitted from having them to advise and support alongside the digital platform. The relationship between nurses and patients improved and this allowed patients to build trust with nurses. However, some patients reported anxiety due to being monitored by nurses constantly and conflicting advice from the nurses and their usual healthcare provider.
Irfan Khan et al. [59]	Qualitative (focus groups) Canada	Patients with multiple chronic conditions and social complexity Providers (healthcare professionals, GPs, nurses) Total: *n* = 18 Patients: *n* = 12 Providers: *n* = 6 Age: 58	Components: mobile platform linked to web‐based portal system, involving goal setting to develop and track self‐management goals and a system to notify providers of hospital visits Setting: primary care and digital Duration: 4 weeks Delivery: platform informed by healthcare professionals; digitally; individually Follow‐up: completion of intervention	N/A	Experiences and expectations of patients and providers around the use of the digital tool	Patients would prefer the tool to be adapted to manage their conditions and behaviours holistically to reflect the broader contextual factors which influence the ability to self‐manage and often felt discouraged due to limited progress towards goals. Patients wanted the tool to supplement appointments, but providers believed it could reduce the need for in person appointments.
McCallum et al. [60]	Qualitative (interviews) United Kingdom	Two or more long‐term conditions, aged 30 to 65 Total: *n* = 14 Males: *n* = 6 Females: *n* = 8 Age: 40 to 63 Conditions: 5	*Same intervention as Mercer2016* [47] Components: whole‐system primary care intervention to allow longer consultations with patients to identify patient priorities, support self‐management and generate a care plan Setting: primary care Duration: 12 months with 3 consultations on average per patient Delivery: led by HCPs; in‐person; individually Follow‐up: interviewed at different time points	N/A	Experiences of the CARE plus intervention	Mixed experiences were reported from participants. Participants benefitted from the longer consultations with professionals as they felt listened to. Although participants were signposted to relevant services, some did not use them. The self‐management resources pack was beneficial to some participants, but some did not use them or did not remember receiving them.
Ngangue et al. [61]	Qualitative (interviews) Canada	Patients with three or more chronic conditions; healthcare professionals; family members of patients Total: *n* = 30 HCPs: *n* = 16 Patients: *n* = 9 Family: *n* = 5 Age: 20 to 72 Conditions: 3 to 6	*Same intervention as Fortin2021* [64] Components: patient‐centred care, self‐management support, interprofessional collaboration and motivational interviewing, involving an individualised care plan developed in accordance with patients' goals Setting: primary care Duration: 4 months, with visits dependent on patient needs Delivery: led by HCPs; in‐person; individually Follow‐up: interview 6 months after starting the intervention	N/A	Perceptions and experiences of the intervention	The intervention was useful from the perspective of healthcare professionals, patients and caregivers. Challenges identified included the availability and time involved in relocating health professionals.
Ngangue et al. [62]	Qualitative (interviews) Canada	Managers and healthcare professionals who delivered the intervention Total: *n* = 29 Age: 20 to 69	*Same intervention as Fortin2021* [64] Components: patient‐centred care, self‐management support, interprofessional collaboration and motivational interviewing, involving an individualised care plan developed in accordance with patients' goals Setting: care primary care Duration: 4 months, with visits dependent on patient needs Delivery: led by HCPs; in‐person; individually Follow‐up: interview 6 months after starting the intervention	N/A	Facilitators and barriers reported by HCPs who were involved in the intervention or its implementation	The interviews identified facilitators to the implementation of this intervention including familiarity with intervention components and focus on what the patient needed and wanted. Barriers included organisational and external factors affecting the intervention, lack of resources, high staff turnover and communication difficulties.

Abbreviations: con = control group, EQ‐5D = EuroQol‐5D, EQ‐VAS = EuroQol visual analogue scale, GPs = general practitioners, HCPs = heath care professionals, int = intervention group, MLTCs = multiple long‐term conditions, N/A = not applicable, NHS = National Health Service.

**Table 3:** Mixed‐methods studies. Summary of the characteristics of mixed‐methods studies based on study design, population, intervention, outcomes and study findings.

References	Study design country	Population	Intervention	Comparator	Primary and secondary outcome(s)	Key findings
Fisher et al. [63]	Mixed methods (RCT and focus groups) Canada	At least 3 chronic conditions, aged 65 years or over Total: *n* = 59 Intervention: *n* = 30 Control: *n* = 29 Males: *n* = 13 (con); *n* = 17 (int) Females: *n* = 16 (con); *n* = 13 (int) Age: 65–69 = 3; 70–74 = 8; 75 + = 19 (int) 65–69 = 2; 70–74 = 6; 75 + = 21 (con) Conditions: 8.63 (int); 8.72 (con)	Components: patient centred care to individualise care plans according to priorities, team collaboration within primary care, coordination of care to support care plans with regular screening and assessment of risk factors, support self‐management and behavioural change. Setting: community based Duration: 6 months with monthly in home visits Delivery: led by HCPs; in‐person; individually Follow‐up: baseline and 6 months	Usual care (home care services as per community care access guidelines)	Primary outcome: health‐related quality of life Secondary outcomes: mental functioning; depressive symptoms; anxiety; self‐efficacy; healthcare and social service use and costs. To investigate barriers and facilitators to intervention using focus groups	No significant improvements in health‐related quality of life or in any of the secondary outcomes. Focus groups with providers highlighted that team collaboration and communication was beneficial to providers and the approach enabled empowerment of participants. However, the small sample size often resulted in poor attendance from the providers at discussions regarding patients.
Fortin et al. [64]	Mixed methods (RCT and interviews) Canada	Three or more chronic conditions, aged 18 to 80 Total: *n* = 284 Intervention: *n* = 144 Control: *n* = 140 Males: *n* = 63 (con); *n* = 69 (int) Females: *n* = 77 (con); *n* = 75 (int) Age: 60.8 ± 10.6 (int) 61.1 ± 10.3 (con) Conditions: 5.0 (int); 5.0 (con)	Components: Patient‐centred care, self‐management support, interprofessional collaboration and motivational interviewing, involving an individualised care plan developed in accordance with patients' goals Setting: primary care Duration: 4 months, with visits dependent on patient needs Delivery: led by HCPs; in‐person; individually Follow‐up: baseline and 4 months	Usual care (including elective or urgent appointments)	Primary outcomes: self‐management; self‐efficacy Secondary outcomes: health status; quality of life; psychological distress; health behaviours	Neutral effects across the domains of self‐management, with a statistically significant improvement in the self‐monitoring and insight domain. Physical activity and healthy eating improved significantly, but health status and quality of life were unchanged. Qualitative findings indicated that participants felt they improved in self‐efficacy, quality of life and health status, although this was not observed in the quantitative findings.
O'Toole et al. [65]	Mixed methods (before‐after design and focus groups) Ireland	Two or more conditions, age 18 or over Total: *n* = 16 Males: *n* = 4 Females: *n* = 12 Age: 66.5 Conditions: 4.0	*Same intervention as Garvey2015* [43] Components: included occupational therapy, peer support in group meetings and goal setting based on prioritisation of patient preferences; focused on self‐management, fatigue and energy management, managing mental health, physical activity, healthy eating, managing medications, communication and goal setting. Setting: primary care Duration: 6 weeks, with a session held weekly Delivery: led by occupational therapists; in‐person; group Follow‐up: baseline, immediately post‐intervention and 8 weeks	No control group	Primary outcomes: occupational performance; ability to perform daily activities; participation in social and community activities; depression and anxiety; self‐efficacy; quality of life Secondary outcomes: perceptions of impact, perception and delivery of intervention	The intervention improved perceptions of and satisfaction with occupational performance, frequency of activity participation and self‐efficacy. Qualitative findings corroborated the quantitative findings, with participants benefitting from the group setting and social interaction.
Ryan et al. [66]	Mixed methods (before‐after design and focus groups/interviews) Ireland	At least two chronic conditions, aged 18 or over Total: *n* = 19 Males: *n* = 5 Females: *n* = 14 Age: Site A 57.7 ± 9.1; Site B 66 ± 59.9 Conditions: Site A 3.1 ± 1.1; Site B 3.5 ± 1.1 Medications: Site A 4.7 ± 3.0; Site B 5.2 ± 2.7	Components: combination of tailored exercise and self‐management support education sessions, including physical activity, prioritising, pain management, sleep, stress and emotional well‐being, nutrition, goal setting and lifestyle balance and signposting Setting: community (leisure centres) Duration: 6 weeks, with weekly sessions Delivery: led by physiotherapists and fitness instructors, group sessions in person Follow‐up: baseline and immediately on completion	No control group	Primary outcomes: feasibility of the intervention Secondary outcomes: quality of life, self‐efficacy, experiences of intervention	The programme improved quality of life, self‐efficacy and physical activity scores. Qualitative findings corroborated the quantitative findings with patients and facilitators identifying significant benefits of the intervention both psychologically and mentally and they valued the group setting, duration and frequency of the intervention sessions.
Skou et al. [67]	Mixed methods (before‐after design and interviews) Denmark	Two or more chronic conditions Facilitators of the intervention Total: *n* = 20 (11 took part in interviews) Age: 67.1 ± 6.9 Females: *n* = 8; Males: *n* = 12 Number of conditions: 9.2 ± 4.0 Interviews: People with MLTC: *n* = 11 Facilitators: *n* = 2	Components: personalised, supervised exercise therapy and self‐management support programme, using goal setting, supervised exercise and home‐based exercise sessions. Setting: home based and department at hospital Duration: 12 weeks, with weekly sessions Delivery: led by physiotherapists and HCPs, with individual and group sessions in person Follow‐up: baseline and 3 months post intervention	No control group	Primary outcomes: health related quality of life; self‐rated health; physical function; experiences of intervention Secondary outcomes: adverse and serious adverse events; compliance	Seven out of twenty participants had clinically relevant improvements in quality of life. Nine out of twenty participants had improvements in function. Fifty percent had compliance with supervised exercise sessions and 70% showed compliance with self‐management support sessions. Participants and facilitators valued the individualised and holistic approach to MLTCs. Being in a group allowed participants to feel safe and supported by others. There were mixed responses to the supervised exercise sessions, as some participants had no issues with the sessions whereas others lacked time and motivation to take part in the sessions.

Abbreviations: con = control group, EQ‐5D = EuroQol‐5D, EQ‐VAS = EuroQol visual analogue scale, GPs = general practitioners, HCPs = heath care professionals, int = intervention group, MLTCs = multiple long‐term conditions, N/A = not applicable, NHS = National Health Service.

### 3.2 Participants

A total of 5888 participants were included in the studies (54.9% females). The average age of participants in quantitative studies ranged from 52 to 78 years and from 40 to 92 years in qualitative studies. Only five of twenty‐eight studies (17.8%) reported data on ethnicity. Eleven studies (39.3%) reported socioeconomic data from participants.

The mean number of conditions ranged from two to nine. The specific conditions present within the population were reported in 19 studies, with the most common being cardiovascular disease (*n* = 15, 53.6%), diabetes (*n* = 14, 50.0%) and respiratory conditions (*n* = 13, 46.4%).

### 3.3 Intervention Characteristics

The self‐management support interventions were often complex and involved multiple different approaches and components. Nineteen studies reported a theory or framework which was used to the guide the development of the self‐management interventions (67.9%), with the Medical Research Council Framework for Complex Interventions the most commonly reported (*n* = 7, 25.0%) (Table 4).

**Table 4:** Components of self‐management support interventions. Summary of the theories and components used in the self‐management support interventions, based on the eight self‐management components detailed by Barlow et al. [12]. Table adapted from Okpako et al. [68].

References	Theory and/or framework used in development of intervention	Self‐management components based on Barlow et al. [12]							
		Information	Drug management	Symptom management	Management of psychological consequences	Lifestyle changes	Social support	Communication	Other (e.g., action plans, problem solving, goal setting)
Boult et al. [40]	Not detailed	Yes: education and support provided to individuals and family caregivers	No	Yes: individuals monitored by HCPs on a monthly basis	No	No	No	No	Yes: HCPs created an action plan based on comprehensive health assessment. HCPs helped co‐ordinate health providers and transitions of care. HCPs facilitated access to community resources. Use of motivational interviewing to promote self‐management.
Contant et al. [41]	Not detailed	Yes: health education provided to individuals on health conditions, chronic disease management and risk factors, such as obesity, physical inactivity and smoking. Supported by provision of printed material and information.	Yes: counselling on medication	No	No	Yes: individuals were able to meet HCPs in the disciplines of physical activity, nutrition or smoking cessation	No	No	Yes: person‐centred motivation interviewing approach to self‐management support. Individualised action plan designed between nurses and individuals. Interdisciplinary collaboration across disciplines of nursing, respiratory therapy, smoking cessation, nutrition, and physical activity.
Doyle et al. [58]	Not detailed	Yes: individuals were able to view education materials related to their conditions and how to use the monitoring devices on the digital health platform	No	Yes: digital platform consisted of sensing devices to measure symptoms, such as blood pressure, blood glucose, blood oxygen levels and breathlessness. Triage nurses were able to view and respond to any alerts (e.g., if thresholds are breached).	Yes: digital platform allowed for mood to be monitored	Yes: individuals were able to set goals in relation to physical activity	Yes: individuals were able to add people to the digital platform as part of their care network and share information with them	No	No
Fisher et al. [63]	Bandura's Social Cognitive Theory; Medical Research Council Framework for Complex Interventions	No	No	No	No	No	Yes: HCPs engaged informal caregivers and supporting them in their role	No	Yes: support for self‐management using a strengths‐based approach which allowed the individual to address challenges and achieve personal goals through improvement of self‐efficacy. Holistic approach focused on prevention and health promotion using a care plan which accounted for all factors which impact self‐management (e.g., social determinants of health, social support). Interdisciplinary collaboration and communication which valued the separate knowledge of different HCPs, individuals and caregivers.
Fortin et al. [64]	Chronic Care Model; Patient‐Centred Clinical Model	No	No	No	No	No	No	No	Yes: person‐centred care approach, interdisciplinary collaboration and motivational interviewing to create an individualised action plan based on individual's goals. Individuals were then referred to the most appropriate professional(s) matching the individual goals.
Fortin et al. [42]	Chronic Care Model; Patient‐Centred Clinical Model	No	No	No	No	No	No	No	Yes: person‐centred care approach, interdisciplinary collaboration and motivational interviewing to create an individualised action plan based on individual's goals. Individuals were then referred to the most appropriate professional(s) matching the individual goals.
Garvey et al. [43]	Bandura's Theory of Self‐Efficacy	Yes: self‐management education was provided to individuals in weekly group sessions	Yes: sessions covered managing medications	Yes: sessions focused on fatigue and energy management	Yes: sessions covered managing stress and anxiety and maintaining mental health and wellbeing	Yes: sessions covered keeping physically active and health eating	Yes: group sessions encouraged peer support	Yes: sessions covered how to communicate effectively with HCPs	Yes: use of goal setting based on individual preferences
Hwang et al. [44]	Individual and Family Self‐Management Theory	Yes: self‐management education was provided on the digital platform using educational videos and daily assignments (such as quizzes or photo‐uploading), alongside in‐person group education sessions	Yes: themes included medication adherence	Yes: themes included sleep and stress management and oral health; digital health programme promoted self‐monitoring of medication, diet, physical activity and sleep through regular reminders	Yes: themes included stress management	Yes: themes included physical activity and healthy diet	Yes: group sessions encouraged peer support	No	Yes: use of individualised health coaching to support goal setting and personalised feedback; themes included supporting problem solving
Irfan Khan et al. [59]	Individual and Family Self‐Management Theory	No	No	Yes: Goals focused on physical wellbeing, mobility and pain management	Yes: goals focused on mental wellbeing (e.g., mood, memory)	Yes: Goals focused on diet or weight management	Yes: Goals covered social well‐being	No	Yes: Use of a mobile, web‐based portal system to develop and track self‐management goals
Khunti et al. [45]	Social Learning Theory	Yes: Sessions involved education and addressing key non‐disease‐specific self‐management challenges and themes	Yes: sessions covered managing treatments	Yes: Promoted person‐centred self‐monitoring with use of reminder and motivational text messages to support long‐term changes	Yes: sessions covered managing emotions	Yes: sessions primarily focused on increasing physical activity	Yes: Group sessions encouraged peer support	Yes: Sessions covered how to communicate effectively with HCPs	Yes: Sessions comprised of goal setting
Lear et al. [46]	Not detailed	No	Yes: individuals regularly completed a questionnaire related to medication adherence	Yes: individuals routinely completed a symptom report about disease‐specific symptoms, biometric data (e.g., weight, blood pressure, blood glucose). Alerts were generated on the digital health platform if targets were not met and discussions held between nurses and individuals	Yes: Individuals regularly completed a questionnaire related to mood or presence of depression.I	Yes: individuals regularly completed a questionnaire related to diet, physical activity and smoking habits	No	No	Yes: co‐development of an action plan between nurse and individual to set goals and targets
McCallum et al. [60]	Medical Research Council Framework for Complex Interventions	Yes: Provision of self‐management support materials	No	No	Yes: provision of mindfulness‐based stress management CDs and cognitive behavioural therapy self‐help booklet.	No	No	No	Yes: HCPs encouraged to carry out holistic, person‐centred assessments to identify individual's concerns and priorities and co‐develop an action plan and set goals. HCPs encouraged to link individuals with community resources.
Mercer et al. [47]	Medical Research Council Framework for Complex Interventions	Yes: Provision of self‐management support materials	No	No	Yes: provision of mindfulness‐based stress management CDs and cognitive behavioural therapy self‐help booklet	No	No	No	Yes: HCPs encouraged to carry out holistic, person‐centred assessments to identify individual's concerns and priorities and co‐develop an action plan and set goals. HCPs encouraged to link individuals with community resources.
Ngangue et al. [61]	Chronic Care Model; Patient‐Centred Clinical Model	No	No	No	No	No	No	No	Yes: Person‐centred care approach, interdisciplinary collaboration and motivational interviewing to create an individualised action plan based on individual's goals. Individuals were then referred to the most appropriate professional(s) matching the individual goals.
Ngangue et al. [62]	Chronic Care Model; Patient‐Centred Clinical Model	No	No	No	No	No	No	No	Yes: Person‐centred care approach, interdisciplinary collaboration and motivational interviewing to create an individualised action plan based on individual's goals. Individuals were then referred to the most appropriate professional(s) matching the individual goals.
O'Toole et al. [65]	Medical Research Council Framework for Complex Interventions	Yes: Self‐management education was provided to individuals in weekly group sessions	Yes: Sessions covered managing medications	Yes: Sessions focused on fatigue and energy management	Yes: Sessions covered managing stress and anxiety and maintaining mental health and well‐being	Yes: Sessions covered keeping physically active and health eating	Yes: group sessions encouraged peer support	Yes: sessions covered how to communicate effectively with HCPs	Yes: Use of goal setting based on individual preferences
O'Toole et al. [48]	Bandura's Theory of Self‐Efficacy	Yes: Self‐management education was provided to individuals in weekly group sessions	Yes: Sessions covered managing medications	Yes: Sessions focused on fatigue and energy management	Yes: Sessions covered managing stress and anxiety and maintaining mental health and well‐being	Yes: Sessions covered keeping physically active and health eating	Yes: Group sessions encouraged peer support	Yes: sessions covered how to communicate effectively with HCPs	Yes: Use of goal setting based on individual preferences
Panagioti et al. [49]	Not detailed	Yes: provision of information and motivation for long‐term condition management	No	No	Yes: Low intensity support for low mood, including assessment for common mental health problems and behavioural techniques to manage mood	Yes: Use of health coaching to promote healthy behaviours around diet, exercise, smoking and alcohol	No	No	Yes: Use of social prescribing to link to community resources or local support
Park et al. [50]	Not detailed	Yes: provision of group education on self‐management behaviours	No	No	No	Yes: use of group exercise sessions to enhance body movements	Yes: Group sessions encouraged peer support	No	Yes: Individualised counselling for goal setting and encouragement of self‐management behaviours
Pellet et al. [51]	Cumulative Complexity Model; Theoretical Framework to Guide Patient/Family Teaching	Yes: Provision of self‐management education based on the activation level of the individuals (e.g., on warning signs, health behaviours)	Yes: self‐management education comprised of medication plans and following appointments	No	No	No	No	Yes: Education included who to contact if needed (e.g., HCPs, hospital).	Yes: education provided was individualised based on needs and priorities
Reed et al. [52]	Not detailed	No	Yes: Individualised care plan could comprise of medication management	No	Yes: Use of cognitive behavioural therapy and motivational interviewing to manage low mood	No	Yes: individualised care plan could address psychosocial and/or carer problems	No	Yes: Collaborative approach between HCPs and individuals to identify problems, set goals and develop individual care plans
Ryan et al. [66]	Medical Research Council Framework for Complex Interventions; Bandura's Theory of Self‐Efficacy	Yes: Self‐management education sessions were provided to groups on a weekly basis	No	Yes: Education comprised of pain management and sleep hygiene	Yes: Education comprised of stress management and emotional wellbeing	Yes: Sessions included supervised exercise and physical activity. Education comprised of nutrition and diet information.	Yes: group sessions encouraged peer support	No	Yes: Education comprised of prioritisation and pacing information and goal setting. Signposting to local resources occurred throughout the programme.
Skou et al. [67]	Medical Research Council Framework for Complex Interventions	Yes: Self‐management education to improve self‐management skills and equip individuals with knowledge on their conditions. Included provision of educational materials and videos.	No	No	No	Yes: Programme included supervised exercise sessions and home‐based exercise sessions. Information was provided to support diet.	Yes: group sessions encouraged peer support	No	Yes: collaborative approach between physiotherapist and individual to set goals and develop individualised action plan. Helped individuals identify problems and support them in overcoming these challenges.
Skou et al. [53]	Medical Research Council Framework for Complex Interventions	Yes: Self‐management support sessions to improve self‐management skills and motivation	No	Yes: Sessions included education on symptoms, pain management and self‐monitoring	Yes: sessions included education on mental health, coping strategies and mindfulness	Yes: Programme included supervised exercise sessions, involving strengthening and balance. Information was provided to support diet.	Yes: Sessions covered involving carers and relatives. Group sessions encouraged peer support.	Yes: Sessions covered how to communicate effectively with HCPs	Yes: Sessions covered goal setting and self‐care
Sommers et al. [54]	Not detailed	No	No	Yes: Individuals were monitored regularly by nurses and social workers	No	No	No	No	Yes: use of interdisciplinary collaboration to set a care plan for individuals and families. This involved setting goals and encouraging self‐management. Nurses promoted use of community services or local support.
Struwe et al. [55]	Not detailed	Yes: Self‐management education provided to improve knowledge, skills and confidence needed to manage health conditions and maintain self‐management behaviours	Yes: Intervention promoted self‐management skills related to medication management	Yes: Intervention promoted self‐management skills related to personal health record development and red flag identification	No	No	No	No	Yes: intervention aimed to promote self‐management through goal setting and action planning
Williams‐Livingston et al. [56]	Community‐Based Participatory Research Conceptual Model	No	No	No	No	No	No	No	Yes: Development of an individualised action plan in collaboration between community health workers and individuals, encouraging and support self‐management. Involved linkages to community support and community engagement activities.
Yang et al. [57]	Information‐Motivation‐Behavioural Skills model	Yes: provision of education related to importance of taking medication and development medication self‐management skills	Yes: Medication self‐management programme to enhance medication adherence	No	No	No	No	No	Yes: nurses used motivational interviewing to help individuals change negative attitudes towards medications. This involved developing an individualised action plan for improving medication self‐management capacity and adherence.

Abbreviation: HCPs = healthcare professionals.

The most common intervention components identified were goal setting (*n* = 20, 71.4%) and individualised action plans (*n* = 16, 57.1%). Interventions commonly focused on supporting mental health and emotional wellbeing (*n* = 14, 50.0%), promoting lifestyle changes, such as diet or physical activity (*n* = 14. 50.0%) or on management of multiple medications (*n* = 11, 39.3%). In contrast, few interventions focused on common symptoms of MLTCs, such as pain (*n* = 3, 10.7%) or fatigue management (*n* = 3, 10.7%).

### 3.4 Outcomes

**Figure 2:**
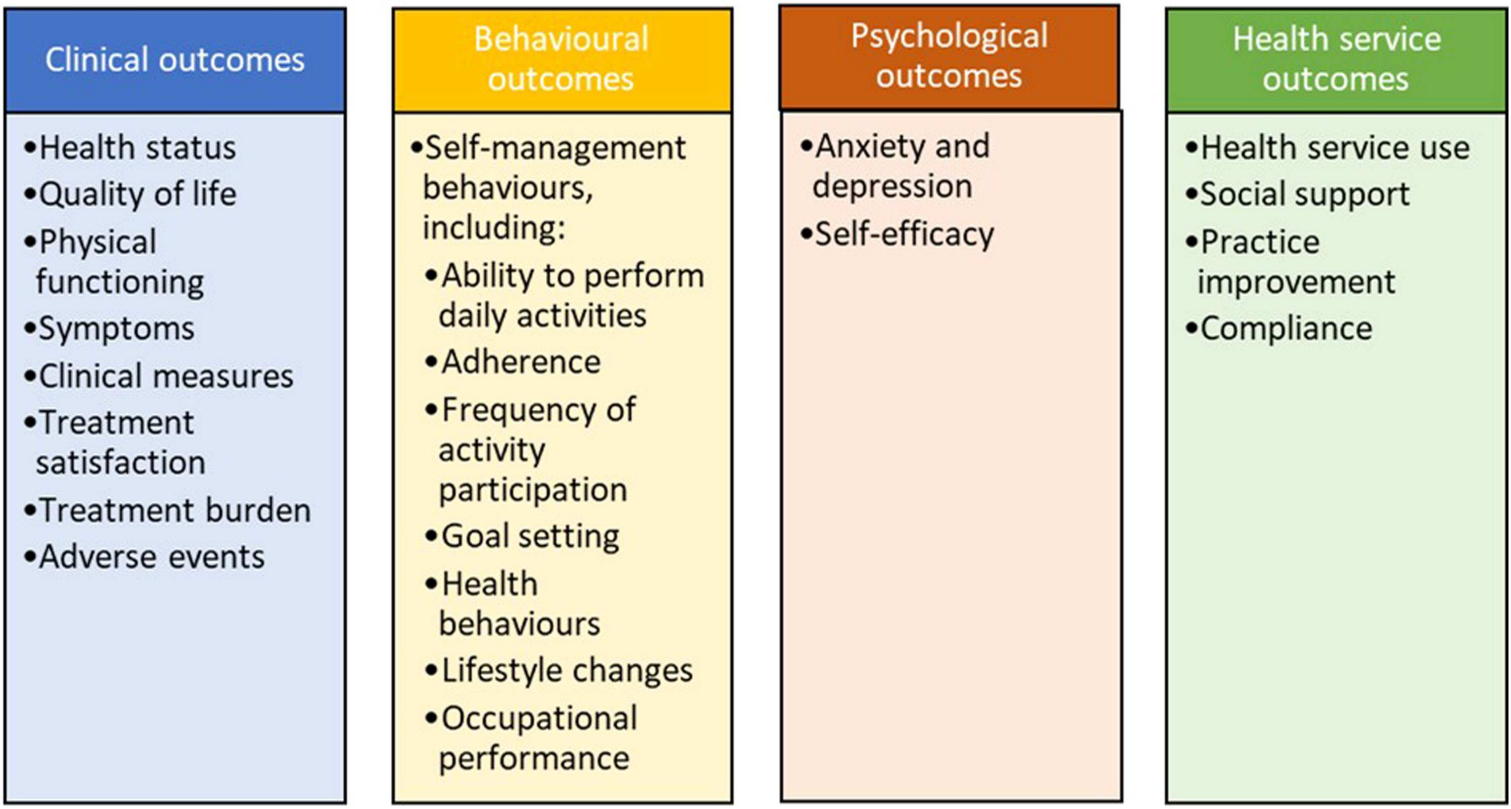
Outcomes used in the included studies. List of outcomes used in the included studies categorised into four groups; clinical, behavioural, psychological and health service outcomes.

A range of outcomes were used to assess interventions (Figure 2).

### 3.5 Quality Appraisal

The quality of the studies varied. For RCTs, seven were considered good quality, five had poor quality and three were assessed as unclear (Table 5). The two before‐after studies (Table 6) and cross‐sectional study (Table 7) were poor quality. The five qualitative studies (Table 8) and five mixed methods studies (Table 9) were good quality.

**Table 5:** Quality assessment for randomised controlled trials. Quality assessment for 15 randomised controlled trials using the Critical Appraisal Skills Programme (CASP) checklist for randomised controlled trials. Overall quality ratings (good/unclear/poor) were determined through discussion between two reviewers, based on the number and importance of criteria met for each appraisal tool.

References	Title	CASP randomised controlled trial standard checklist													Overall quality
		CASP01	CASP02	CASP03	CASP04a (participants)	CASP04b (providers)	CASP04c (outcome assessors)	CASP05	CASP06	CASP07	CASP08	CASP09	CASP10	CASP11	
Boult et al. [40]	The effect of guided care teams on the use of health services: Results from a cluster‐randomized controlled trial	Yes	Yes	Can't tell	No	No	Can't tell	Yes	Yes	Yes	Yes	Yes	Can't tell	Can't tell	Poor
Contant et al. [41]	A multidisciplinary self‐management intervention among patients with multimorbidity and the impact of socioeconomic factors on results	Yes	Yes	Yes	No	No	Can't tell	Yes	Yes	Yes	Yes	Yes	Yes	Can't tell	Good
Garvey et al. [43]	OPTIMAL, an occupational therapy led self‐management support programme for people with multimorbidity in primary care: A randomized controlled trial	Yes	Yes	Yes	No	No	No	Yes	Yes	Yes	Yes	Yes	Yes	Can't tell	Good
Hwang et al. [44]	Effectiveness of a digital health coaching self‐management programme for older adults living alone with multiple chronic conditions: A randomized controlled trial	Yes	Yes	Yes	No	No	Yes	Yes	Yes	Yes	Yes	Yes	Yes	Yes	Good
Khunti et al. [45]	Promoting physical activity with self‐management support for those with multimorbidity: A randomised controlled trial	Yes	Yes	Yes	No	No	Yes	Yes	Yes	Yes	Yes	No	Yes	No	Unclear
Lear et al. [46]	Assessment of an Interactive Digital Health‐Based Self‐management Programme to Reduce Hospitalizations Among Patients With Multiple Chronic Diseases: A Randomized Clinical Trial	Yes	Yes	Yes	No	Yes	Yes	Yes	Yes	Yes	Yes	Yes	Yes	Can't tell	Good
Mercer et al. [47]	The CARE Plus study—A whole‐system intervention to improve quality of life of primary care patients with multimorbidity in areas of high socioeconomic deprivation: Exploratory cluster randomised controlled trial and cost‐utility analysis	Yes	Yes	Yes	No	No	Yes	Yes	Yes	Yes	Yes	Yes	Yes	Can't tell	Good
O'Toole et al. [48]	Effect of the OPTIMAL programme on self‐management of multimorbidity in primary care: A randomised controlled trial	Yes	Yes	Yes	No	No	Yes	Yes	Yes	Yes	Yes	Can't tell	Yes	Can't tell	Unclear
Panagioti et al. [49]	Is telephone health coaching a useful population health strategy for supporting older people with multimorbidity? An evaluation of reach, effectiveness and cost‐effectiveness using a ‘trial within a cohort'	Yes	Yes	Yes	No	No	Can't tell	Yes	Yes	Yes	Yes	Can't tell	Yes	Can't tell	Unclear
Park et al. [50]	Effect of a health coaching self‐management programme for older adults with multimorbidity in nursing homes	Yes	Yes	Can't tell	No	No	Yes	Can't tell	Yes	Yes	No	Yes	No	Can't tell	Poor
Reed et al. [52]	A self‐management support programme for older Australians with multiple chronic conditions: A randomised controlled trial	Yes	Yes	Yes	Yes	No	Can't tell	Yes	Yes	Yes	Yes	Yes	Yes	Can't tell	Good
Skou et al. [53]	Exercise therapy and self‐management support for individuals with multimorbidity: A randomized and controlled trial	Yes	Yes	Yes	No	No	Yes	Yes	Yes	Yes	Yes	Yes	Yes	Yes	Good
Sommers et al. [54]	Physician, nurse and social worker collaboration in primary care for chronically ill seniors	Yes	Can't tell	Can't tell	Can't tell	Can't tell	Can't tell	No	Yes	Yes	Yes	Yes	Yes	Can't tell	Poor
Struwe et al. [55]	Changes in Patient Activation in a Self‐Management Intervention	Yes	Yes	Yes	Can't tell	Can't tell	Can't tell	Can't tell	Yes	Yes	No	Can't tell	Can't tell	Can't tell	Poor
Yang et al. [57]	Effects of a nurse‐led medication self‐management intervention on medication adherence and health outcomes in older people with multimorbidity: A randomised controlled trial	Yes	Yes	Yes	No	No	Yes	No	Yes	Yes	Yes	Yes	No	Can't tell	Poor

*Note:* CASP01: Did the study address a clearly focused research question? CASP02: Was the assignment of participants to interventions randomised? CASP03: Were all participants who entered the study accounted for at its conclusion? CASP04a: Were the participants ‘blind’ to intervention they were given? CASP04b: Were the investigators ‘blind’ to the intervention they were giving to participants? CASP04c: Were the people assessing/analysing outcome/s ‘blinded’? CASP05: Were the study groups similar at the start of the randomised controlled trial? CASP06: Apart from the experimental intervention, did each study group receive the same level of care (i.e., were they treated equally)? CASP07: Were the effects of intervention reported comprehensively? CASP08: Was the precision of the estimate of the intervention or treatment effect reported? CASP09: Do the benefits of the experimental intervention outweigh the harms and costs? CASP10: Can the results be applied to your local population/in your context? CASP11: Would the experimental intervention provide greater value to the people in your care than any of the existing interventions?

**Table 6:** Quality assessment for before‐after studies. Quality assessment for two before‐after studies using the National Heart, Lung, and Blood Institute (NHLBI) quality assessment for before‐after studies. Overall quality ratings (good/unclear/poor) were determined through discussion between two reviewers, based on the number and importance of criteria met for each appraisal tool.

References	Title	NHLBI quality assessment tool for pre‐post studies with no control group												Overall quality
		NHLBI01	NHLBI02	NHLBI03	NHLBI04	NHLBI05	NHLBI06	NHLBI07	NHLBI08	NHLBI09	NHLBI10	NHLBI11	NHLBI12	
Fortin et al. [42]	One year follow‐up and exploratory analysis of a patient‐centred interdisciplinary care intervention for multimorbidity	Yes	Yes	Yes	Cannot determine	No	Yes	Yes	No	No	Yes	No	Cannot determine	Poor
Pellet et al. [51]	Improving patient activation with a tailored nursing discharge teaching intervention for multimorbid inpatients: A quasi‐experimental study	Yes	Yes	Yes	Yes	Cannot determine	Yes	Yes	No	No	Yes	No	Yes	Poor

*Note:* NHLBI01: Was the study question or objective clearly stated? NHLBI02: Were eligibility/selection criteria for the study population prespecified and clearly described? NHLBI03: Were the participants in the study representative of those who would be eligible for the test/service/intervention in the general or clinical population of interest? NHLBI04: Were all eligible participants that met the prespecified entry criteria enroled? NHLBI05: Was the sample size sufficiently large to provide confidence in the findings? NHLBI06: Was the test/service/intervention clearly described and delivered consistently across the study population? NHLBI07: Were the outcome measures prespecified, clearly defined, valid, reliable, and assessed consistently across all study participants? NHLBI08: Were the people assessing the outcomes blinded to the participants' exposures/interventions? NHLBI09: Was the loss to follow‐up after baseline 20% or less? Were those lost to follow‐up accounted for in the analysis? NHLBI10: Did the statistical methods examine changes in outcome measures from before to after the intervention? Were statistical tests done that provided *p* values for the pre‐to‐post changes? NHLBI11: Were outcome measures of interest taken multiple times before the intervention and multiple times after the intervention (i.e., did they use an interrupted time‐series design)? NHLBI12: If the intervention was conducted at a group level (e.g., a whole hospital, a community, etc.) did the statistical analysis take into account the use of individual‐level data to determine effects at the group level?

**Table 7:** Quality assessment for cross‐sectional studies. Quality assessment for one cross‐sectional study using the National Heart, Lung, and Blood Institute (NHLBI) quality assessment for cross‐sectional studies. Overall quality ratings (good/unclear/poor) were determined through discussion between two reviewers, based on the number and importance of criteria met for each appraisal tool.

References	Title	NHLBI quality assessment tool for observational cohort and cross‐sectional studies														Overall quality
		NHLBI01	NHLBI02	NHLBI03	NHLBI04	NHLBI05	NHLBI06	NHLBI07	NHLBI08	NHLBI09	NHLBI10	NHLBI11	NHLBI12	NHLBI13	NHLBI14	
Williams‐Livingston et al. [56]	Community‐Based Participatory Research in Action: The Patient‐Centred Medical Home and Neighbourhood	Yes	Yes	No	Can't tell	No	No	No	N/A	Yes	No	No	Can't tell	No	No	Poor

*Note:* NHLBI01: Was the study question or objective clearly stated? NHLBI02: Was the study population clearly specified and defined? NHLBI03: Was the participation rate of eligible persons at least 50%? NHLBI04: Were all the subjects selected or recruited from the same or similar populations (including the same time period)? Were inclusion and exclusion criteria for being in the study prespecified and applied uniformly to all participants? NHLBI05: Was a sample size justification, power description, or variance and effect estimates provided? NHLBI06: For the analyses in this paper, were the exposure(s) of interest measured prior to the outcome(s) being measured? NHLBI07: Was the timeframe sufficient so that one could reasonably expect to see an association between exposure and outcome if it existed NHLBI08: For exposures that can vary in amount or level, did the study examine different levels of the exposure as related to the outcome (e.g., categories of exposure, or exposure measured as continuous variable)? NHLBI09: Were the exposure measures (independent variables) clearly defined, valid, reliable and implemented consistently across all study participants? NHLBI10: Was the exposure(s) assessed more than once over time? NHLBI11: Were the outcome measures (dependent variables) clearly defined, valid, reliable and implemented consistently across all study participants? NHLBI12: Were the outcome assessors blinded to the exposure status of participants? NHLBI13: Was loss to follow‐up after baseline 20% or less? NHLBI14: Were key potential confounding variables measured and adjusted statistically for their impact on the relationship between exposure(s) and outcome(s)?

**Table 8:** Quality assessment for qualitative studies. Quality assessment for five qualitative studies using the Critical Appraisal Skills Programme (CASP) checklist for qualitative studies. Overall quality ratings (good/unclear/poor) were determined through discussion between two reviewers, based on the number and importance of criteria met for each appraisal tool.

References	Title	CASP qualitative standard checklist										Overall quality
		CASP01	CASP02	CASP03	CASP04	CASP05	CASP06	CASP07	CASP08	CASP09	CASP10	
Doyle et al. [58]	The role of phone‐based triage nurses in supporting older adults with multimorbidity to digitally self‐manage—Findings from the ProACT proof‐of‐concept study	Yes	Yes	Yes	Yes	Yes	No	Yes	Yes	Yes	Author discussed findings in terms of practical implications and in terms of an existing framework	Good
Irfan Khan et al. [59]	mHealth Tools for the Self‐Management of Patients With Multimorbidity in Primary Care Settings: Pilot Study to Explore User Experience	Yes	Yes	Yes	Yes	Yes	No	Yes	Yes	Yes	Authors discussed how this research would influence future work exploring the use of electronic self‐management tools and how refinement and evaluation of electronic tools could better assist self‐management of chronic conditions	Good
McCallum et al. [60]	Exploring the utility of self‐determination theory in complex interventions in multimorbidity: A qualitative analysis of patient experiences of the CARE Plus intervention	Yes	Yes	Yes	Yes	Yes	No	Yes	Yes	Yes	Authors discussed their findings in the context of the wider literature and the implications of using the self‐determination theory to explore the experiences of self‐management interventions	Good
Ngangue et al. [61]	Patients, caregivers and health‐care professionals' experience with an interdisciplinary intervention for people with multimorbidity in primary care: A qualitative study	Yes	Yes	Yes	Yes	Yes	No	Yes	Yes	Yes	Authors discussed their findings in the context of wider literature and the focus of future research and interventions	Good
Ngangue et al. [62]	Evaluating the implementation of interdisciplinary patient‐centred care intervention for people with multimorbidity in primary care: A qualitative study	Yes	Yes	Yes	Yes	Yes	No	Yes	Yes	Yes	Authors discussed findings in the context of the literature and implications	Good

*Note:* CASP01: Was there a clear statement of the aims of the research? CASP02: Is a qualitative methodology appropriate? CASP03: Was the research design appropriate? CASP04: Was the recruitment strategy appropriate? CASP05: Were the data collected in a way that addressed the research issue? CASP06: Has the relationship between researcher and participants been adequately considered? CASP07: Have ethical issue been taken into consideration? CASP08: Was the data analysis sufficiently rigorous? CASP09: Is there a clear statement of findings? CASP10: How valuable is the research?

**Table 9:** Quality assessment for mixed methods studies. Quality assessment for five mixed methods studies using the mixed methods appraisal tool (MMAT). Overall quality ratings (good/unclear/poor) were determined through discussion between two reviewers, based on the number and importance of criteria met for each appraisal tool.

References	Title	Mixed methods appraisal tool (MMAT)				
		MMAT01	MMAT02	MMAT03	MMAT04	MMAT05
Fisher et al. [63]	Self‐management programme versus usual care for community‐dwelling older adults with multimorbidity: A pragmatic randomized controlled trial in Ontario, Canada	Yes	Yes	Yes	Yes	Yes
Fortin et al. [64]	Scaling up patient‐centred interdisciplinary care for multimorbidity: A pragmatic mixed‐methods randomized controlled trial	Yes	Yes	Yes	Yes	Yes
O'Toole et al. [65]	Impact of an occupation‐based self‐management programme on chronic disease management	Yes	Yes	Yes	Yes	Yes
Ryan et al. [66]	Development and feasibility of an inter‐agency physical activity and education programme for adults with multimorbidity in primary care: Activ8	Yes	Yes	Yes	Yes	Yes
Skou et al. [67]	Personalised exercise therapy and self‑management support for people with multimorbidity: Feasibility of the MOBILIZE intervention	Yes	Yes	Yes	Yes	Yes

*Note:* MMAT01: Is there adequate rationale for using a mixed methods design to address the research question? MMAT02: Are the different components of the study effectively integrated to answer the research question? MMAT03: Are the outputs of the integration of qualitative and quantitative components adequately interpreted? MMAT04: Are divergences and inconsistencies between quantitative and qualitative results adequately addressed? MMAT05: Do the different components of the study adhere to the quality criteria of each tradition of the methods involved?

### 3.6 Quantitative Synthesis

Data were available to statistically pool in a meta‐analysis for quality of life (*n* = 4), self‐efficacy (*n* = 6), and self‐management behaviours (*n* = 3). Twenty‐one studies were included in the narrative synthesis to include vote counting based on direction of effect (Tables S1–S4) [34, 35].

### 3.7 Primary Outcomes

#### 3.7.1 Quality of Life

**Figure 3:**
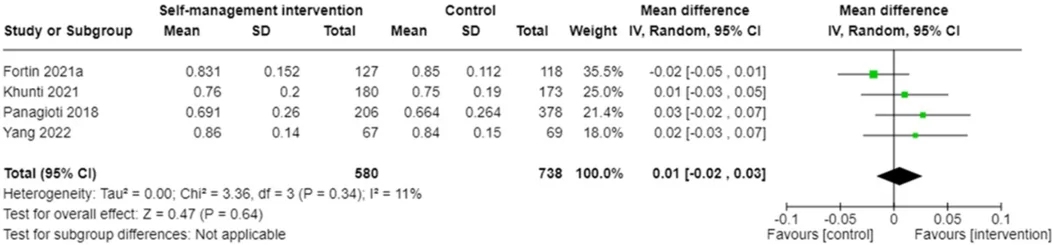
Meta‐analysis of quality of life outcomes. Effect estimates and Forest plot for quality of life, using the EQ‐5D questionnaire.

The meta‐analysis indicated no effect of self‐management support interventions on quality of life (*n* = 4; MD = 0.01; 95% CI: −0.02–0.03; *p* = 0.64; *I* ^2^ = 11%; Figure 3). In addition, 13 studies used quality of life as an outcome measure, but could not be included in the meta‐analysis, Across these studies, findings were mixed (Table S1), with six studies reporting improvements in quality of life, four reporting no change and three reporting a negative impact on quality of life.

#### 3.7.2 Psychological Outcomes

**Figure 4:**
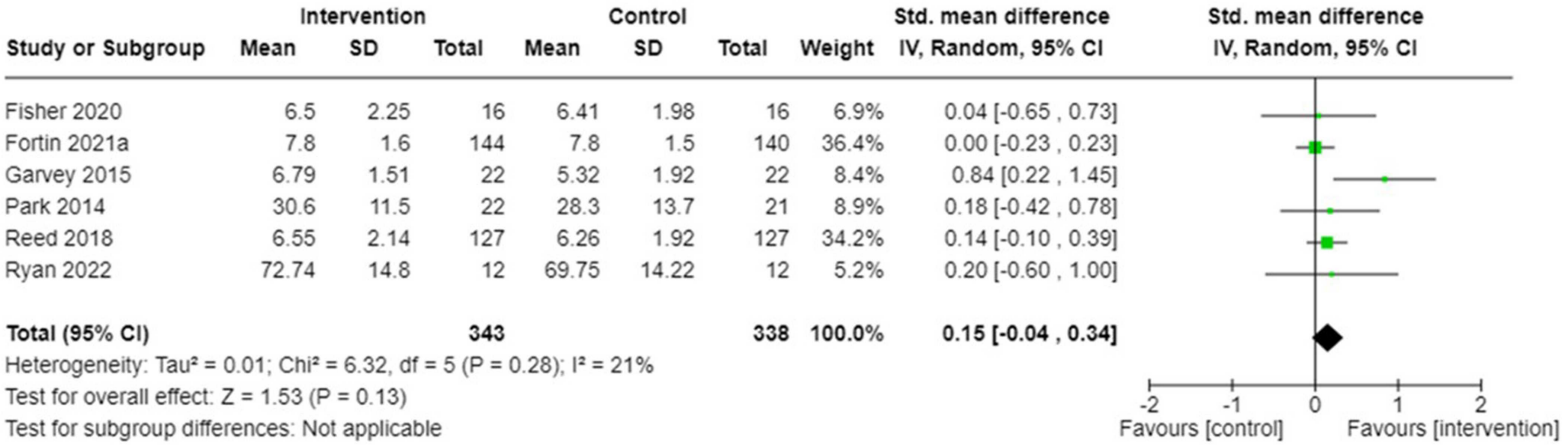
Meta‐analysis of self‐efficacy outcomes. Effect estimates and Forest plot for self‐efficacy.

In terms of psychological outcomes, the meta‐analysis indicated no clear evidence of an effect of self‐management interventions on self‐efficacy (*n* = 6; SMD = 0.15; 95% CI: −0.04–0.34; *p* = 0.13, *I* ^2^ = 21%; Figure 4). In contrast, based on vote counting and direction of effect, 6 studies showed improvements in self‐efficacy which favoured intervention groups compared with the control group (Table S1). One study showed mixed to no changes in self‐efficacy.

Mental health outcomes, including anxiety and depression were included in 12 studies (Table S1). Mixed findings were observed across these outcomes. Eight studies reported outcomes which favoured the intervention groups whereas four studies reported no changes.

### 3.8 Secondary Outcomes

Secondary outcomes reported in the studies included behavioural, clinical and health service outcomes.

#### 3.8.1 Behavioural Outcomes

**Figure 5:**
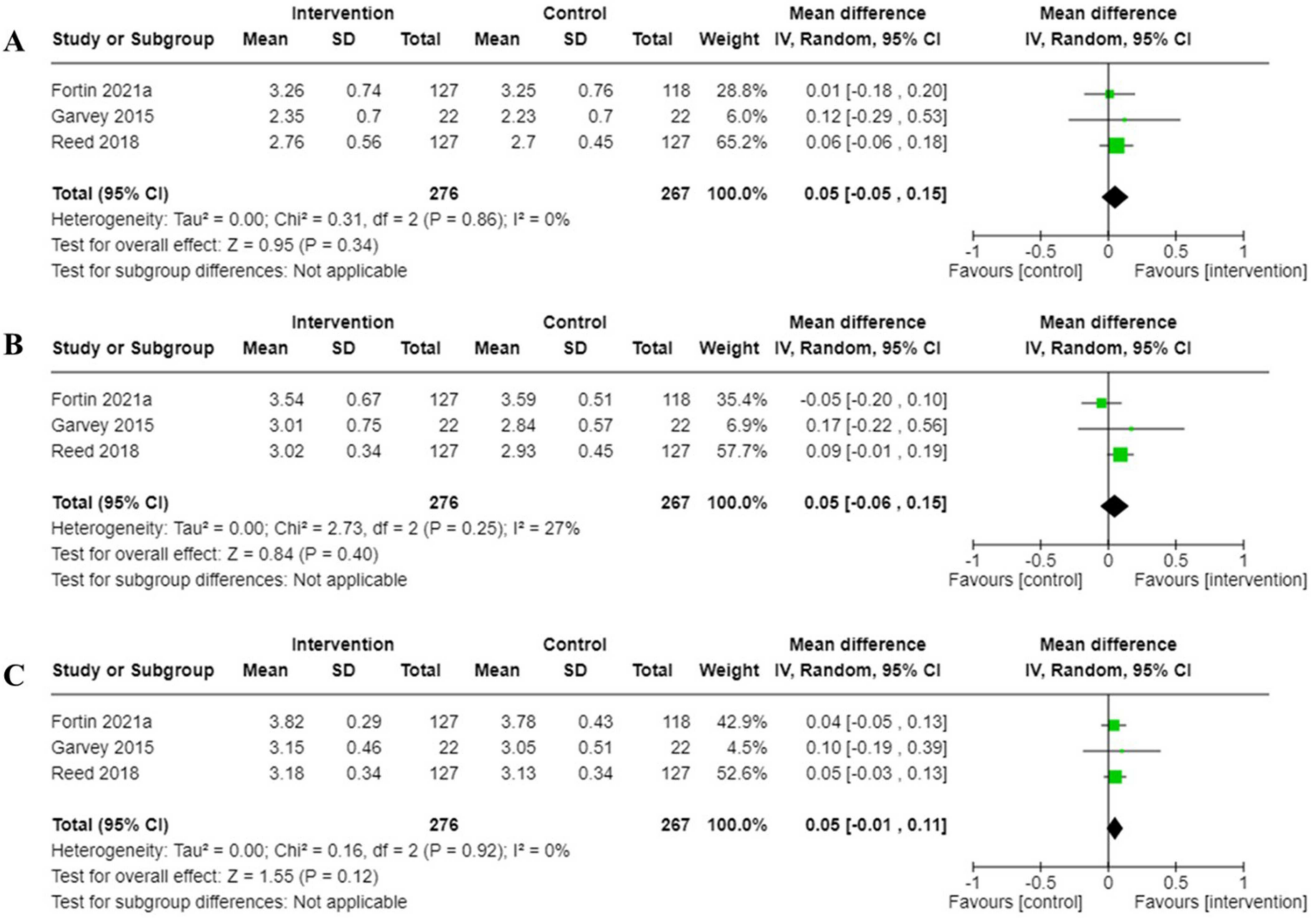
Meta‐analysis of self‐management behaviour outcomes. (A) Effect estimates and Forest plot for emotional wellbeing domain of self‐management, using the Health Education Impact Questionnaire (HeiQ); (B) Effect estimates and Forest plot for social integration and support domain of self‐management, using the HeiQ; (C) Effect estimates and Forest plot for health service navigation domain of self‐management, using the HeiQ.

**Figure 6:**
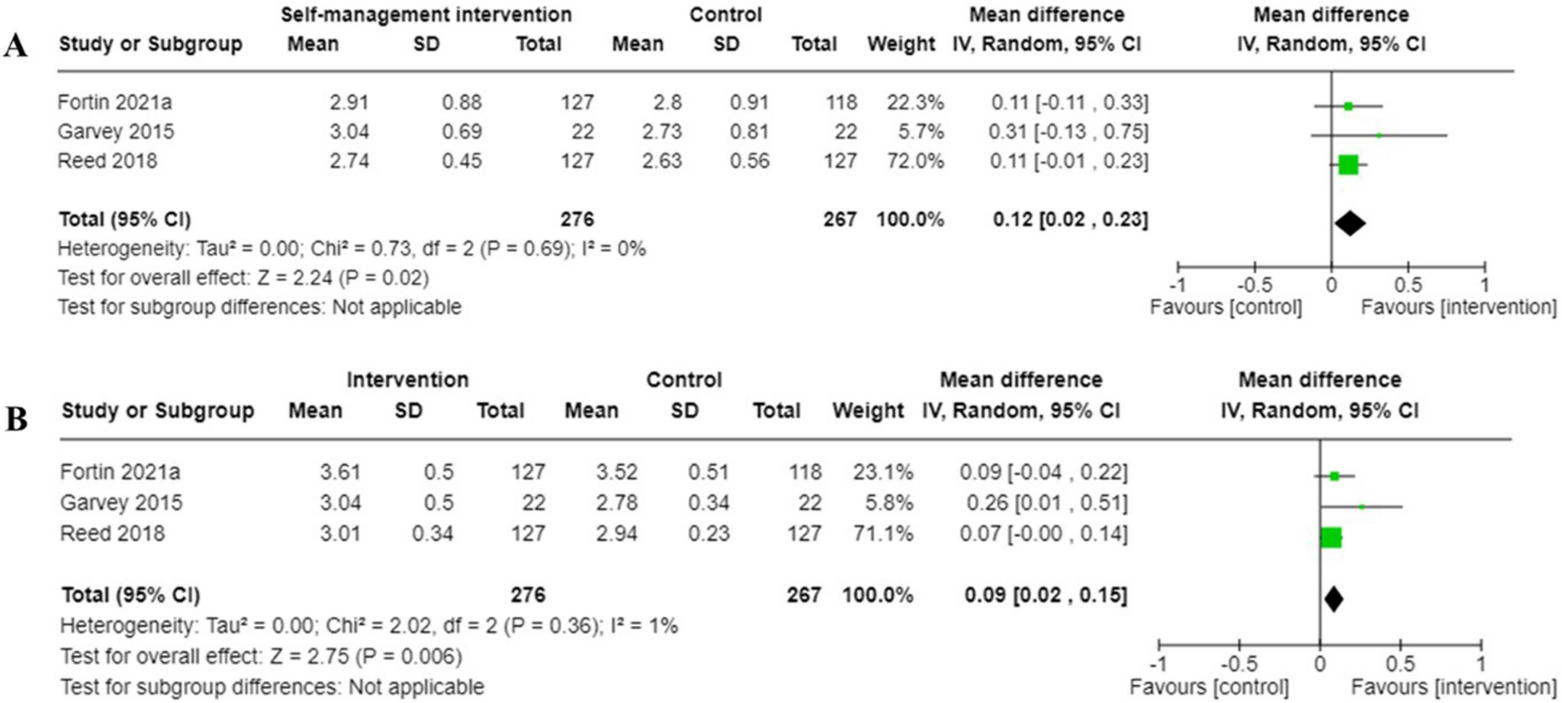
Meta‐analysis of self‐management behaviour outcomes. (A) Effect estimates and Forest plot for health directed behaviour domain of self‐management, using the Health Education Impact Questionnaire (HeiQ); (B) Effect estimates and Forest plot for skill and technique acquisition domain of self‐management, using the HeiQ.

**Figure 7:**
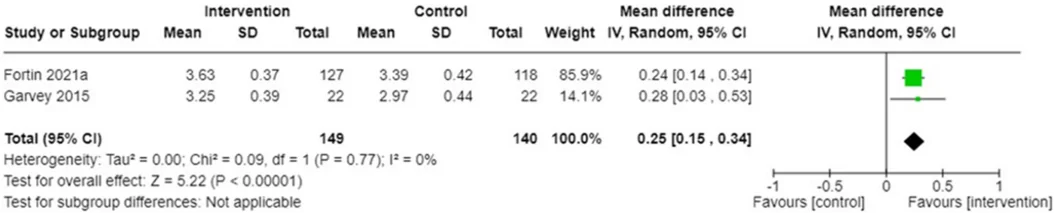
Meta‐analysis of self‐management behaviour outcomes. Effect estimates and Forest plot for self‐monitoring and insight domain of self‐management, using Health Education Impact Questionnaire (HeiQ).

Both meta‐analysis and vote counting based on direction of effect indicated mixed findings for an intervention effect on self‐management behaviours (Figures 5–7 and Table S2). There was no evidence of an intervention effect across three domains of self‐management: emotional well‐being (*n* = 3; MD = 0.05; 95% CI: −0.05–0.15; *p* = 0.34; *I* ^2^ = 0%; Figure 5A); social integration and support (n = 3; MD = 0.05; 95% CI: −0.06–0.15; *p* = 0.40; *I* ^2^ = 27%; Figure 5B); and health service navigation (*n* = 3; MD = 0.05; 95% CI: −0.01–0.11; *p* = 0.12; *I* ^2^ = 0%; Figure 5C).

Pooled analysis of three studies (Figure 6) indicated a statistically significant effect favouring self‐management interventions on the health‐directed behaviour domain of self‐management (*n* = 3; MD = 0.12; 95% CI: 0.02–0.23; *p* = 0.02; *I* ^2^ = 0%, Figure 6A) and on the skill and technique acquisition domain of self‐management (*n* = 3; MD = 0.09; 95% CI: 0.02–0.15; *p* = 0.006; *I* ^2^ = 1%, Figure 6B).

Pooled analysis of three studies indicated a statistically significant effect favouring self‐management interventions on the self‐monitoring and insight domain of self‐management (*n* = 3; MD = 0.17; 95% CI: 0.01–0.33; *p* = 0.03). However, a considerable level of heterogeneity was detected (*I* ^2^ = 81%). When Reed et al. [52] was removed from the meta‐analysis, heterogeneity was reduced and the pooled analysis of the two studies remained significant (*n* = 2; MD = 0.25; 95% CI: 0.15–0.34; *p* < 0.00001; *I* ^2^ = 0%; Figure 7).

Eleven studies used self‐management behaviours as outcome measures and were included in the effect direction plot (Table S2). Seven studies showed mixed to no effects on self‐management behaviours in the intervention group. Only four studies showed positive improvements in self‐management favouring the intervention group,

Additional behavioural outcomes were also assessed across studies and were included in the effect direction plot (Table S2). Improvements in ability to perform daily activities, frequency of activity participation and occupational performance were observed (*n* = 3) (Table S2). However, there were mixed to no changes observed in lifestyle behaviours (*n* = 5), engagement in social activities (*n* = 3) and goal attainment (*n* = 2) (Table S2).

#### 3.8.2 Clinical Outcomes

For clinical outcomes, mixed findings were observed in health status (*n* = 6), specific clinical measures including blood measures and symptoms (*n* = 4), medication adherence (*n* = 3) and physical functioning (*n* = 2) (Table S3). No changes were observed in treatment burden (*n* = 1).

#### 3.8.3 Health Service Use

Ten studies had health service use as an outcome, including general practitioner visits, emergency department visits and hospital outpatient visits. Overall, mixed findings in health service use were observed (Table S4).

### 3.9 Qualitative Synthesis

Ten studies were included in the thematic synthesis, consisting of five qualitative studies and five mixed‐method studies. These studies involved interviewing HCPs, people with MLTCs, general practice managers and family and caregivers. Thematic synthesis resulted in five analytical themes which are reported below, with example quotations provided in Table S5.

### 3.10 Theme 1: Interdisciplinary Collaboration Can Facilitate Better Support

This theme highlights the importance of communication, especially between HCPs across different healthcare disciplines. Communication between HCPs as part of interdisciplinary collaboration facilitated the delivery of care and support interventions. Interdisciplinary team meetings were seen as beneficial as this enabled in‐depth discussion regarding patient care and more efficient follow up of patients.

HCPs also valued the input from other disciplines, such as from personal support workers, as they were able to provide a different point of view on the delivery of care and support. In addition, HCPs also identified that the sharing of data and information related to patients' care between professionals facilitated care and support.

### 3.11 Theme 2: Influential External and Contextual Factors

A range of external and contextual factors at an individual and organisational level influenced the delivery of support and engagement in self‐management interventions.

#### 3.11.1 Health, Personal and Social Challenges

Living with the symptom burden of MLTCs negatively impacted engagement in self‐management interventions, with mental health issues and pain specifically identified as barriers to self‐management support. Individuals also faced a number of personal and social challenges which negatively affected their ability to engage in support interventions. Individuals had other priorities beyond their own health, including social issues. For example, some individuals were at risk of homelessness, had financial issues or could not access adequate transport.

#### 3.11.2 Therapeutic, Social and Family Relationships

Support networks and therapeutic relationships could positively and negatively influence delivery and engagement in self‐management support.

Positive therapeutic relationships were important for effective support. Individuals valued good communication and longer consultations with HCPs. However, some individuals reported a poor relationship with HCPs, which influenced what was discussed at consultations and impacted the support received.

Strong social and family relationships positively influenced engagement in self‐management support activities and interventions. Some individuals also reported that peer support positively impacted experiences with self‐management support interventions, particularly if the programmes were in a group‐based setting. However, poor social and family networks negatively impacted experiences of self‐management support programmes due to feelings of isolation.

#### 3.11.3 Organisation Challenges

External factors at an organisational level, such as a health system reorganisation, impacted the delivery of the self‐management support intervention. For example, one study [62] reported that there was a reorganisation to integrate health and social service centres occurring at the same time as the implementation of the intervention.

### 3.12 Theme 3: Importance of Individualisation and Empowerment

Individuals commonly reported feeling empowered by the support received and this improved motivation to engage in further self‐management activities. HCPs observed empowerment of individuals due to the approach and components used as part of the self‐management interventions. Maintaining autonomy for individuals was important and this allowed them to take an active role in their own care, as noted by HCPs and caregivers. Self‐management support tasks, such as setting realistic goals, also enabled empowerment of individuals as successful achievement of goals improved motivation and encouraged positive behaviours.

### 3.13 Theme 4: ‘Real World’ Implementation

#### 3.13.1 Barriers to Implementation and Support Delivery

Factors were identified by participants which negatively impacted the delivery of support. Specific limitations and issues with components of support interventions were identified by HCPs, including concerns related to referring individuals to other clinicians or disciplines as part of interdisciplinary collaboration. HCPs also faced practical issues with implementing the support programmes. Some lacked resources, such as physical space, staff and finances, whilst others lacked the necessary time needed to meet with other clinicians and attend consultations.

The complexity of the self‐management support interventions also posed challenges, as HCPs found some intervention concepts difficult to understandand and explain to patients. Implementation was further complicated if the intervention did not fit with existing principles or frameworks which were currently being used as part of routine care.

#### 3.13.2 Facilitators to Implementation and Support Delivery

In contrast, factors were also identified which improved the delivery and implementation of self‐management support interventions. HCPs identified that the provision of resources, such as leadership teams, easier access to other health disciplines, finances or suitable training in the intervention concepts, were important to facilitate implementation of support programmes.

Some HCPs appreciated the approaches used in the self‐management support interventions, including the person‐centred approach or focus on MLTCs instead of single conditions These approaches aligned with their principles as healthcare providers. HCPs who had previous experience with the intervention concepts, such as interdisciplinary collaboration or motivational approaches, found it easier to implement and deliver support.

Individuals receiving the support interventions appreciated the design and structure of the interventions, including the length and setting, and provision of regular feedback and follow‐up with HCPs. Learning self‐management strategies, such as fatigue and medication management, was seen as highly valuable by individuals.

### 3.14 Theme 5: Actual and Perceived Positive Outcomes

Individuals reported positive improvements across a range of outcomes following participation in the interventions. Individuals commonly reported improved confidence, motivation and self‐management behaviours. Improved motivation was found to lead to increased engagement in social and daily activities and attendance at health appointments. Individuals also reported improvements in physical health, mental health and quality of life. Other individuals reported positive changes in lifestyle behaviours, such as dietary habits and exercise. Self‐management support also led to positive changes in individuals' outlook on their own health.

### 3.15 Meta‐Integration

Quantitative synthesis predominately focused on outcome‐related data and did not include data related to factors which impacted the delivery and experiences of the self‐management interventions. Therefore, the meta‐integration is focused on integrating quantitative and qualitative data related to outcomes of self‐management interventions. Of the 10 outcomes included for the meta‐integration of quantitative and qualitative data, 7 were refuted and 3 complemented, based on authors' interpretation [39, 69] (Table 10).

**Table 10:** Table summarising meta‐integration of quantitative and qualitative data. Table summarising meta‐integration of qualitative and quantitative findings on outcomes and how the findings complemented or refuted each other.

Concept	Quantitative synthesis	Qualitative synthesis	Integration
Quality of life	There was no statistically significant improvement in quality of life in the self‐management intervention group compared with control group	Descriptive theme(s): ‘Support led to improvements in health outcomes’; ‘Moderate improvement in health’; ‘Support was beneficial to those who received it’ Analytical theme(s): ‘Actual and Perceived Positive Outcomes’	The quantitative data on quality of life outcomes was **refuted** by the qualitative synthesis, which found that individuals reported improved physical health and quality of life as a result of the support intervention received
Mental health	Narrative synthesis indicated that mixed findings were reported across psychological outcomes. This ranged from improvements in psychological outcomes to no change in outcomes.	Descriptive themes(s): ‘Support led to improvements in mental health outcomes’; ‘Support led to positive perceptions of their health’ Analytical theme(s): ‘Actual and Perceived Positive Outcomes’	The quantitative data on mental health outcomes was **refuted** by the qualitative synthesis, which found that individuals reported improvements in their mental health and positive changes in the perceptions of their own health, such as improved expectations of living with multiple conditions
Self‐efficacy	There was no statistically significant effect in self‐efficacy in the self‐management intervention group compared with control group	Descriptive theme(s): ‘Support led to improvements in behavioural outcomes’ Analytical theme(s): ‘Actual and Perceived Positive Outcomes’	The quantitative data on self‐efficacy outcomes was **refuted** by the qualitative synthesis, which found that healthcare providers and individuals reported improved confidence and motivation to self‐manage their health conditions
Self‐management behaviours	There was a statistically significant improvement in health directed behaviour in the self‐management intervention group	Descriptive theme(s): ‘Support led to improvements in behavioural outcomes’ Analytical theme(s): ‘Actual and Perceived Positive Outcomes’	The quantitative data on health directed self‐management behaviours was **complemented** by the qualitative synthesis which found that individuals and healthcare professionals reported improvements in self‐management behaviours, due to increased motivation resulting from the interventions
	There was no statistically significant effect on emotional well‐being in the self‐management intervention group	Descriptive themes(s): ‘Support led to improvements in mental health outcomes’; ‘Support led to positive perceptions of their health’ Analytical theme(s): ‘Actual and Perceived Positive Outcomes’	The quantitative data on emotional well‐being outcomes was **refuted** by the qualitative synthesis, which found that individuals reported improvements in their mental health and positive changes in the perceptions of their own health, such as improved expectations of living with multiple conditions
	There was a statistically significant improvement in self‐monitoring and insight	Descriptive theme(s): ‘Support led to improvements in behavioural outcomes’ Analytical theme(s): 'Actual and Perceived Positive Outcomes’	The quantitative data on self‐monitoring and insight behaviours was **complemented** by the qualitative synthesis which found that individuals and healthcare professionals reported improvements in self‐management behaviours, due to increased confidence in their ability to solve problems and monitor their conditions
	There was a statistically significant improvement in skill and technique acquisition	Descriptive theme(s): ‘Support included learning strategies for self‐management’ Analytical theme(s): “‘Real World’ Implementation”	The quantitative data on skill and technique acquisition outcomes was **complemented** by the qualitative synthesis, which found that individuals valued learning strategies for self‐management, including medication management and stress management strategies
	There was no statistically significant effect in social integration and support in the self‐management group compared with the control group	Descriptive theme(s): ‘Support received improved engagement in social and daily activities’ Analytical theme(s): ‘Actual and Perceived Positive Outcomes’	The quantitative data on social integration and support was **refuted** by the qualitative synthesis, which found that individuals increased engagement in social and daily activities due to improved motivation
	There was no statistically significant effect in health service navigation in the self‐management group compared with the control group	Descriptive themes(s): Support motivated people to engage with healthcare services Analytical theme(s): ‘Actual and Perceived Positive Outcomes’	The quantitative data on health service navigation was **refuted** by the qualitative synthesis, which found that individuals reported increased attendance at health appointments and signposted services due to improved confidence and motivation, resulting from the support received
Lifestyle changes	Narrative synthesis indicated that there were mixed findings observed across lifestyle behaviours, including healthy eating, physical activity, smoking and alcohol consumption. These ranged from improvements in physical activity and healthy eating to a reduction in rates of exercise.	Descriptive theme(s): ‘Support led to improvements in lifestyle behaviours’ Analytical theme(s): ‘Actual and Perceived Positive Outcomes’	The quantitative data on lifestyle changes was **refuted** by the qualitative synthesis, which found that individuals reported positive changes in lifestyle behaviours, such as dietary habits and physical activity

*Note:* **Complemented**: findings from quantitative and qualitative synthesis added to each other and offered explanations for the observations; **Refuted**: findings from quantitative and qualitative synthesis opposed/contradicted each other.

## 4 Discussion

### 4.1 Summary of Findings

This review used a mixed‐methods approach to synthesise both quantitative and qualitative data on self‐management support interventions targeting MLTCs. Twenty‐eight studies met the eligibility criteria, including 18 quantitative studies, 5 qualitative studies and 5 mixed methods studies.

Quantitative synthesis indicated little to no intervention effect for most outcomes, including quality of life or psychological outcomes. While statistically significant improvements favouring the intervention were observed three domains of self‐management behaviours (health‐directed behaviour, self‐monitoring, skill and technique acquisition), findings across other self‐management behaviours were inconsistent. Importantly, the strength of evidence is limited by the low or moderate quality of several included studies. Thematic synthesis of qualitative data identified factors which both positively and negatively impacted engagement in interventions, including social factors and empowerment of individuals.

Meta‐integration showed that qualitative data complemented meta‐analysis findings for several self‐management behaviours, as this data indicated that improved motivation and confidence led to higher engagement in self‐management. However, qualitative data refuted the quantitative findings in quality of life and psychological outcomes, as participants reported improvements in their mental health and quality of life.

### 4.2 Comparison With Previous Research

High heterogeneity was observed across interventions, including variation in components, theoretical foundations, delivery modes and outcomes. This aligns with the findings from a previous systematic review of patient‐engagement interventions for older adults with complex health needs, which also reported substantial variability [70]. This heterogeneity likely reflects the diverse sociodemographic characteristics, number and types of conditions, and differing levels of severity within MLTCs populations [71].

Unlike single condition interventions that focus on disease‐specific self‐management (e.g., insulin dosing, inhaler technique) [24], the interventions included in this systematic review typically adopted a broader, person‐centred approach by addressing lifestyle behaviours, emotional wellbeing, self‐efficacy and medications. Outcomes also often centred on quality of life, mental health and health behaviours rather than disease‐specific clinical indicators (e.g., HbA1_c_ levels) [72, 73, 74].

While previous reviews have quantitatively appraised the effects of various interventions in people with MLTCs, comorbidities or complex health needs, this review synthesised both quantitative and qualitative data on the effects of self‐management support interventions in adults with MLTCs. This adds depth to the existing evidence related to the experiences of self‐management interventions for people with MLTCs.

Previous systematic reviews have provided mixed quantitative results regarding the effects of interventions for MLTCs. One systematic review [75] evaluated the use of goal‐orientated care against standard care for people with MLTCs showed no effect of goal‐orientated care on quality of life across nine trials (*n* = 3127), nor on healthcare admissions in a sub‐analysis of three trials (*n* = 1340), which aligns with the findings of the current review. A review of complex interventions for people with MLTCs [24] also reported limited effects on quality of life and mental health outcomes, further supporting the findings from the current review. The findings from the current review, in combination with existing evidence, suggest that self‐management support interventions have yet to demonstrate consistent benefits across key outcomes, such as quality of life or mental health, in populations with MLTCs.

### 4.3 Strengths and Limitations

To the best of our knowledge, this is the first systematic review to use a mixed methods approach to synthesise evidence on self‐management support interventions for MLTCs, which allowed for a deeper understanding of the effects and experiences of the intervention. This information is key and is commonly required by stakeholders to develop interventions and guidelines [76].

This systematic review followed a pre‐specified, publicly available protocol. An extensive search across multiple databases was conducted, alongside in‐depth supplementary searches. This review adhered to best practice approaches to all stages and reporting was in accordance with PRISMA guidelines (Table S6). This review also focused on MLTCs instead of defined comorbidities or specific conditions, allowing for greater generalisability of the findings to the growing number of people with MLTCs. Authors of the primary studies were contacted where there were missing data, such as mean values and standard deviation. Although not all authors responded or were able to provide the missing data, this allowed for the retrieval of additional data beyond what was provided in the published literature.

The studies were restricted to English language, as resources were not available for translation. This did not result in the exclusion of relevant studies, which could have introduced bias. The quality of the included studies varied from poor to good quality. Employing multiple quality appraisal tools may limit direct comparability of quality across studies. However, the results from each quality appraisal were clearly reported by study design and we did not exclude studies based on quality ratings. The lack of common outcome measures or absence of relevant data restricted the number of studies which could be included in the meta‐analysis and may reduce the strength of the conclusions drawn. More consistency across methods of reporting data and use of outcome measures for MLTCs would allow for improved comparisons between results from different studies and increased confidence in the findings.

### 4.4 Implications for Future Practice

The thematic synthesis also underscored the importance of social, economic and contextual factors on individuals' capacity to engage in self‐management. HCPs should consider these factors and take a more holistic approach to supporting people with MLTCs, which may involve connecting individuals with social prescribing services, peer support groups or other community resources [77]. The varied impact of the social determinants of health further highlights the need for person‐centred self‐management support aligned with what matters most to each individual.

This review has indicated that individuals living with MLTCs have diverse social and healthcare needs, that can be effectively addressed through interdisciplinary collaboration. Strengthening collaboration across healthcare and allied professionals is important for delivering effective self‐management support for individuals living with MLTCs.

### 4.5 Implications for Future Research

This review has shown that a range of outcomes were used to measure health and behavioural improvements, with most tools validated for use in single conditions. The disparity between the quantitative and qualitative findings for quality of life and mental health outcomes suggests that quantitative measures may not capture the full experience of individuals living with MLTCs. As quality of life and mental health are core outcomes in MLTCs research [78], future research should prioritise validation of questionnaires for MLTCs or the development of MLTCs‐specific tools.

Older populations were commonly targeted by the interventions included in this review, with a mean age ranging between 52 and 78 in quantitative studies. Future studies should include wide range of participants, as MLTCs are prevalent across the life course [79]. In addition, ethnic minority groups are more likely to experience greater burden of MLTCs compared to White populations [80, 81, 82]. However, ethnicity was only reported in five studies. This corroborates with a previous review which highlighted limited reporting and underrepresentation of ethnic minority populations in MLTCs research [83]. Similarly, although individuals living socioeconomically deprived areas are twice as likely to experience MLTCs and commonly experience MLTCs at a younger age [84], only 11 studies reported socioeconomic status. Therefore, future MLTCs research should transparently report sociodemographic information of participants and consider conducting sub‐group analysis to understand how these factors influence outcomes.

The prevalence of MLTCs in low‐and‐middle income countries (LMICs) is comparable to the rates of MLTCs in high income countries (HICs) [4]. However, all studies included were conducted in HICs. Therefore, future research should examine self‐management interventions in LMIC contexts.

## 5 Conclusion

Although some improvements were observed in specific self‐management behaviours, there is little to no evidence that self‐management interventions for MLTCs improve most outcomes, including quality of life and mental health. These results remain uncertain due to mixed findings across qualitative and quantitative studies. The inclusion of both qualitative and quantitative studies has generated evidence on the effect and patient and HCPs' experiences of self‐management interventions, which is essential for clinical decision making. However, the disparity between the quantitative and qualitative findings for quality of life and mental health outcomes suggests that current outcome measures may not fully reflect lived experiences of people with MLTCs. To address this gap, future research should prioritise validating outcome measurement tools, specifically for use in MLTCs populations.

## Conflicts of Interest

The authors declare no conflicts of interest.

## Supporting information


Supporting File

## Data Availability

The data that support the findings of this study are available from the corresponding author upon reasonable request.

## References

[1] K. Khunti , H. Sathanapally , and P. Mountain , “Multiple Long Term Conditions, Multimorbidity, and Co‐Morbidities: We Should Reconsider the Terminology We Use,” BMJ 383 (2023): 2327, 10.1136/bmj.p2327. 37832952

[2] J. J. Miranda , A. Bernabe‐Ortiz , R. H. Gilman , et al., “Multimorbidity at Sea Level and High‐Altitude Urban and Rural Settings: The CRONICAS Cohort Study,” Journal of Comorbidity 9 (2019): 75297, 10.1177/2235042X19875297. PMC8240099 34249770

[3] O. A. Asogwa , D. Boateng , A. Marzà‐Florensa , et al., “Multimorbidity of Non‐Communicable Diseases in Low‐Income and Middle‐Income Countries: A Systematic Review and Meta‐Analysis,” BMJ Open 12 (2022): e049133, 10.1136/bmjopen-2021-049133. PMC8785179 35063955

[4] H. Nguyen , G. Manolova , C. Daskalopoulou , S. Vitoratou , M. Prince , and A. M. Prina , “Prevalence of Multimorbidity in Community Settings: A Systematic Review and Meta‐Analysis of Observational Studies,” Journal of Comorbidity 9 (2019): 2235042X19870934, 10.1177/2235042X19870934. PMC6710708 31489279

[5] I. S.‐S. Ho , A. Azcoaga‐Lorenzo , A. Akbari , et al., “Variation in the Estimated Prevalence of Multimorbidity: Systematic Review and Meta‐Analysis of 193 International Studies,” BMJ Open 12 (2022): e057017, 10.1136/bmjopen-2021-057017. PMC9058768 35487738

[6] A. Kingston , L. Robinson , H. Booth , M. Knapp , C. Jagger , et al., “Projections of Multi‐Morbidity in the Older Population in England to 2035: Estimates From the Population Ageing and Care Simulation (PACSim) Model,” Age and Ageing 47 (2018): 374–380, 10.1093/AGEING/AFX201. 29370339 PMC5920286

[7] A. V. Perruccio , J. N. Katz , and E. Losina , “Health Burden in Chronic Disease: Multimorbidity Is Associated With Self‐Rated Health More Than Medical Comorbidity Alone,” Journal of Clinical Epidemiology 65 (2012): 100–106, 10.1016/j.jclinepi.2011.04.013. 21835591 PMC3217092

[8] M. C. Johnston , C. Black , S. W. Mercer , G. J. Prescott , and M. A. Crilly , “Prevalence of Secondary Care Multimorbidity in Mid‐Life and Its Association With Premature Mortality in a Large Longitudinal Cohort Study,” BMJ Open 10 (2020): e033622, 10.1136/bmjopen-2019-033622. PMC7229982 32371508

[9] U. T. Kadam , J. Uttley , P. W. Jones , and Z. Iqbal , “Chronic Disease Multimorbidity Transitions Across Healthcare Interfaces and Associated Costs: A Clinical‐Linkage Database Study,” BMJ Open 3 (2013): e003109, 10.1136/bmjopen-2013. PMC3717459 23872294

[10] Public Health England , “The Health and Social Care Costs of a Selection of Health Conditions and Multi‐Morbidities” (2020), https://www.gov.uk/government/publications/health-and-social-care-costs-of-a-selection-of-health-conditions.

[11] L. Paukkonen , A. Oikarinen , O. Kähkönen , and P. Kaakinen , “Patient Activation for Self‐Management Among Adult Patients With Multimorbidity in Primary Healthcare Settings,” Health Science Reports 5 (2022): e735, 10.1002/hsr2.735. 35873391 PMC9297377

[12] J. Barlow , C. Wright , J. Sheasby , A. Turner , and J. Hainsworth , “Self‐Management Approaches for People With Chronic Conditions: A Review,” Patient Education and Counseling 48 (2002): 177–187, 10.1016/S0738-3991(02)00032-0. 12401421

[13] J. Corbin and A. Strauss , “Managing Chronic Illness at Home: Three Lines of Work,” Qualitative Sociology 8 (1985): 224–247, 10.1007/BF00989485.

[14] J. Dwarswaard , E. J. M. Bakker , A. van Staa , and H. R. Boeije , “Self‐Management Support From the Perspective of Patients With a Chronic Condition: A Thematic Synthesis of Qualitative Studies,” Health Expectations 19 (2016): 194–208, 10.1111/hex.12346. 25619975 PMC5055271

[15] H. Burd and M. Hallsworth , “Supporting Self‐Management A Guide to Enabling Behaviour Change for Health and Wellbeing Using Person‐and Community‐ Centred Approaches” (2016), http://www.nesta.org.uk/publications/supporting-self-management-guide-enabling-behaviour-change-health-and-wellbeing-using-person-and-community-centred-approaches.

[16] J. Ellis , E. Boger , S. Latter , et al., “Conceptualisation of the ‘Good’ Self‐Manager: A Qualitative Investigation of Stakeholder Views on the Self‐Management of Long‐Term Health Conditions,” Social Science & Medicine (1982) 176 (2017): 25–33, 10.1016/j.socscimed.2017.01.018. 28126586

[17] C. Coughlan and M. Watson , “Managing Multimorbidity: Listening to Patients and Integrated Care,” BMJ 368 (2020): 2020, 10.1136/bmj.m713. 32102799

[18] C. J. M. Whitty , C. MacEwen , A. Goddard , et al., “Rising to the Challenge of Multimorbidity,” BMJ 368 (2020): 1–2, 10.1136/bmj.l6964. PMC7190283 31907164

[19] K. R. Lorig and H. R. Holman , “Self‐Management Education: History, Definition, Outcomes, and Mechanisms,” Annals of Behavioral Medicine 26 (2003): 1–7. 12867348 10.1207/S15324796ABM2601_01

[20] N. Mead and P. Bower , “Patient‐Centredness: A Conceptual Framework and Review of the Empirical Literature,” Social Science & Medicine (1982) 51 (2000): 1087–1110. 11005395 10.1016/s0277-9536(00)00098-8

[21] T. S. Tang , M. Funnell , B. Sinco , et al., “Comparative Effectiveness of Peer Leaders and Community Health Workers in Diabetes Selfmanagement Support: Results of a Randomized Controlled Trial,” Diabetes Care 37 (2014): 1525–1534, 10.2337/dc13-2161. 24722495 PMC4030090

[22] N. Almutairi , H. Hosseinzadeh , and V. Gopaldasani , “The Effectiveness of Patient Activation Intervention on Type 2 Diabetes Mellitus Glycemic Control and Self‐Management Behaviors: A Systematic Review of R CTs,” Primary Care Diabetes 14 (2020): 12–20, 10.1016/j.pcd.2019.08.009. 31543458

[23] S. Dineen‐Griffin , V. Garcia‐Cardenas , K. Williams , and S. I. Benrimoj , “Helping Patients Help Themselves: A Systematic Review of Self‐Management Support Strategies in Primary Health Care Practice,” PLoS One 14 (2019): e0220116, 10.1371/journal.pone.0220116. 31369582 PMC6675068

[24] S. M. Smith , E. Wallace , B. Clyne , F. Boland , and M. Fortin , “Interventions for Improving Outcomes in Patients With Multimorbidity in Primary Care and Community Setting: A Systematic Review,” Systematic Reviews 10 (2021): 271, 10.1186/s13643-021-01817-z. 34666828 PMC8527775

[25] M. Sandelowski , C. I. Voils , and J. Barroso , “Defining and Designing Mixed Research Synthesis Studies,” Research in the Schools: A Nationally Refereed Journal Sponsored by the Mid‐South Educational Research Association and the University of Alabama 13 (2006): 29. 20098638 PMC2809982

[26] J. Briggs Institute , “8.1 Introduction to Mixed Methods Systematic Reviews—JBI Manual for Evidence Synthesis—JBI Global Wiki,” in *JBI Manual Evidence Synthesis* (2022), https://jbi-global-wiki.refined.site/space/MANUAL/4689215/8.1+Introduction+to+mixed+methods+systematic+reviews.

[27] M. J. Page , J. E. McKenzie , P. M. Bossuyt , et al., “The Prisma 2020 Statement: An Updated Guideline for Reporting Systematic Reviews,” Systematic Reviews 10 (2021): 89, 10.1186/S13643-021-01626-4/FIGURES/1. 33781348 PMC8008539

[28] M. Dixon‐Woods , S. Agarwal , D. Jones , B. Young , and A. Sutton , “Synthesising Qualitative and Quantitative Evidence: A Review of Possible Methods,” Journal of Health Services Research & Policy 10 (2005): 45–53, 10.1177/135581960501000110. 15667704

[29] E. Hopwood , F. Curtis , M. Hadjiconstantinou , and K. Khunti , “Effectiveness of Person‐Centred Self‐Management Interventions on Adults With Multiple Long‐Term Conditions: A Mixed Methods Systematic Review,” PROSPERO International Prospective Register of Systematic Reviews (2022), https://www.crd.york.ac.uk/prospero/display_record.php?RecordID=302959.

[30] NIHR , “NIHR Evidence—Multiple Long‐Term Conditions (Multimorbidity): Making Sense of the Evidence—Informative and Accessible Health and Care Research,” (2021), https://evidence.nihr.ac.uk/collection/making-sense-of-the-evidence-multiple-long-term-conditions-multimorbidity/.

[31] Critical Appraisal Skills Programme , “CASP Checklists—Critical Appraisal Skills Programme,” in *CASP—Critical Appraisal Skills Programme* (2022), https://casp-uk.net/casp-tools-checklists/.

[32] National Heart, Lung and Blood Institute , “Study Quality Assessment Tools,” NHLBI, NIH (2021), https://www.nhlbi.nih.gov/health-topics/study-quality-assessment-tools.

[33] McGill Department of Family Medicine , “Mixed Methods Appraisal Tool,” In: Department of Family Medicine (2018), https://www.mcgill.ca/familymed/research/projects/mmat.

[34] M. H. Boon and H. Thomson , “The Effect Direction Plot Revisited: Application of the 2019 Cochrane Handbook Guidance on Alternative Synthesis Methods,” Research Synthesis Methods 12 (2021): 29–33, 10.1002/jrsm.1458. 32979023 PMC7821279

[35] M. Campbell , J. E. McKenzie , A. Sowden , et al., “Synthesis Without Meta‐Analysis (SWiM) in Systematic Reviews: Reporting Guideline,” BMJ 368 (2020): 1–6, 10.1136/bmj.l6890. PMC7190266 31948937

[36] J. J. Deeks , J. P. Higgins , and D. G. Altman , “Chapter 10: Analysing Data and Undertaking Meta‐Analyses,” in Cochrane Handbook for Systematic Reviews of Interventions, ed. J. P. T. Higgins , J. Thomas , J. Chandler , M. Cumpston , T. Li , M. J. Page (Cochrane, 2024), https://training.cochrane.org/handbook/current/chapter-10.

[37] J. Poorolajal and S. Noornejad , “Metaplot: A New Stata Module for Assessing Heterogeneity in a Meta‐Analysis,” PLoS One 16 (2021): e0253341, 10.1371/journal.pone.0253341. 34181682 PMC8238175

[38] J. Thomas and A. Harden , “Methods for the Thematic Synthesis of Qualitative Research in Systematic Reviews,” BMC Medical Research Methodology 8 (2008): 45, 10.1186/1471-2288-8-45. 18616818 PMC2478656

[39] G. A. Whitley , P. Hemingway , G. R. Law , A. W. Jones , F. Curtis , and A. N. Siriwardena , “The Predictors, Barriers and Facilitators to Effective Management of Acute Pain in Children by Emergency Medical Services: A Systematic Mixed Studies Review,” Journal of Child Health Care 25 (2021): 481–503, 10.1177/1367493520949427. 32845710 PMC8422593

[40] C. Boult , L. Reider , B. Leff , et al., “The Effect of Guided Care Teams on the Use of Health Services: Results From a Cluster‐Randomized Controlled Trial,” Archives of Internal Medicine 171 (2011): 460–466, 10.1001/archinternmed.2010.540. 21403043 PMC4450357

[41] É. Contant , C. Loignon , T. Bouhali , J. Almirall , and M. Fortin , “A Multidisciplinary Self‐Management Intervention Among Patients With Multimorbidity and the Impact of Socioeconomic Factors on Results,” BMC Family Practice 20 (2019): 53. 31010425 10.1186/s12875-019-0943-6 PMC6477711

[42] M. Fortin , M. Stewart , J. Almirall , et al., “One Year Follow‐Up and Exploratory Analysis of a Patient‐Centered Interdisciplinary Care Intervention for Multimorbidity,” Journal of Multimorbidity and Comorbidity 11 (2021): 39780, 10.1177/26335565211039780. PMC8606917 34820337

[43] J. Garvey , D. Connolly , F. Boland , and S. M. Smith , “OPTIMAL, an Occupational Therapy Led Self‐Management Support Programme for People With Multimorbidity in Primary Care: A Randomized Controlled Trial,” BMC Family Practice 16 (2015): 59, 10.1186/S12875-015-0267-0. 25962515 PMC4438474

[44] M. Hwang , S. Lee , G. E. Park , and Y.‐H. Park , “Effectiveness of a Digital Health Coaching Self‐Management Program for Older Adults Living Alone With Multiple Chronic Conditions: A Randomized Controlled Trial,” Geriatric Nursing 65 (2025): 103509, 10.1016/j.gerinurse.2025.103509. 40633238

[45] K. Khunti , P. J. Highton , G. Waheed , et al., “Promoting Physical Activity With Self‐Management Support for Those With Multimorbidity: A Randomised Controlled Trial,” British Journal of General Practice 71 (2021): e921–e930, 10.3399/BJGP.2021.0172. PMC8574221 34725044

[46] S. A. Lear , M. Norena , D. Banner , et al., “Assessment of an Interactive Digital Health‐Based Self‐Management Program to Reduce Hospitalizations Among Patients With Multiple Chronic Diseases: A Randomized Clinical Trial,” JAMA Network Open 4 (2021): e2140591, 10.1001/jamanetworkopen.2021.40591. 34962560 PMC12243620

[47] S. W. Mercer , B. Fitzpatrick , B. Guthrie , et al., “The Care Plus Study—A Whole‐System Intervention to Improve Quality of Life of Primary Care Patients With Multimorbidity in Areas of High Socioeconomic Deprivation: Exploratory Cluster Randomised Controlled Trial and Cost‐Utility Analysis,” BMC Medicine 14 (2016): 88, 10.1186/S12916-016-0634-2. 27328975 PMC4916534

[48] L. O'Toole , D. Connolly , F. Boland , and S. M. Smith , “Effect of the OPTIMAL Programme on Self‐Management of Multimorbidity in Primary Care: A Randomised Controlled Trial,” British Journal of General Practice 71 (2021): e303–e311, 10.3399/BJGP20X714185. PMC7959668 33685920

[49] M. Panagioti , D. Reeves , R. Meacock , et al., “Is Telephone Health Coaching a Useful Population Health Strategy for Supporting Older People With Multimorbidity? An Evaluation of Reach, Effectiveness and Cost‐Effectiveness Using a ‘Trial Within a Cohort’,” BMC Medicine 16 (2018): 80, 10.1186/s12916-018-1051-5. 29843795 PMC5975389

[50] Y. H. Park and H. Chang , “Effect of a Health Coaching Self‐Management Program for Older Adults With Multimorbidity in Nursing Homes,” Patient Preference and Adherence 8 (2014): 959–970, 10.2147/PPA.S62411. 25045253 PMC4094628

[51] J. Pellet , M. Weiss , F. Zúñiga , and C. Mabire , “Improving Patient Activation With a Tailored Nursing Discharge Teaching Intervention for Multimorbid Inpatients: A Quasi‐Experimental Study,” Patient Education and Counseling 118 (2024): 108024, 10.1016/j.pec.2023.108024. 37862876

[52] R. L. Reed , L. Roeger , S. Howard , et al., “A Self‐Management Support Program for Older Australians With Multiple Chronic Conditions: A Randomised Controlled Trial,” Medical Journal of Australia 208 (2018): 69–74, 10.5694/mja17.00127. 29385967

[53] S. T. Skou , M. Nyberg , M. Dideriksen , et al., “Exercise Therapy and Self‐Management Support for Individuals With Multimorbidity: A Randomized and Controlled Trial,” Nature Medicine 31 (2025): 3176–3182, 10.1038/s41591-025-03779-4. PMC12443619 40588675

[54] L. S. Sommers , K. I. Marton , J. C. Barbaccia , and J. Randolph , “Physician, Nurse, and Social Worker Collaboration in Primary Care for Chronically Ill Seniors,” Archives of Internal Medicine 160 (2000): 1825. 10871977 10.1001/archinte.160.12.1825

[55] L. A. Struwe , M. S. Schmaderer , and L. Zimmerman , “Changes in Patient Activation in a Self‐Management Intervention,” Western Journal of Nursing Research 42 (2020): 194–200, 10.1177/0193945919848091. 31092139

[56] A. Williams‐Livingston , T. Henry Akintobi , and A. Banerjee , “Community‐Based Participatory Research in Action: The Patient‐Centered Medical Home and Neighborhood,” Journal of Primary Care & Community Health 11 (2020): 68456, 10.1177/2150132720968456. PMC7682305 33203309

[57] C. Yang , D. T. F. Lee , X. Wang , and S. Y. Chair , “Effects of a Nurse‐Led Medication Self‐Management Intervention on Medication Adherence and Health Outcomes in Older People With Multimorbidity: A Randomised Controlled Trial,” International Journal of Nursing Studies 134 (2022): 104314, 10.1016/j.ijnurstu.2022.104314. 35849886

[58] J. Doyle , P. McAleer , C. Van Leeuwen , et al., “The Role of Phone‐Based Triage Nurses in Supporting Older Adults With Multimorbidity to Digitally Self‐Manage—Findings From the ProACT Proof‐of‐Concept Study,” Digital Health 8 (2022): 205520762211311, 10.1177/20552076221131140. PMC9551328 36238753

[59] A. Irfan Khan , A. Gill , C. Cott , P. K. Hans , and C. Steele Gray , “mHealth Tools for the Self‐Management of Patients With Multimorbidity in Primary Care Settings: Pilot Study to Explore User Experience,” JMIR mHealth and uHealth 6 (2018): e171, 10.2196/mhealth.8593. 30154073 PMC6134226

[60] M. McCallum , C. M. Gray , P. Hanlon , R. O'Brien , and S. W. Mercer , “Exploring the Utility of Self‐Determination Theory in Complex Interventions in Multimorbidity: A Qualitative Analysis of Patient Experiences of the CARE Plus Intervention,” Chronic illness 17 (2021): 433–450, 10.1177/1742395319884106. 31674216

[61] P. A. Ngangue , C. Forgues , T. Nguyen , et al., “Patients, Caregivers and Health‐Care Professionals' Experience With an Interdisciplinary Intervention for People With Multimorbidity in Primary Care: A Qualitative Study,” Health Expectations: An International Journal of Public Participation in Health Care and Health Policy 23 (2020): 318–327, 10.1111/hex.13035. 32035012 PMC7104629

[62] P. Ngangue , J. B. Brown , C. Forgues , et al., “Evaluating the Implementation of Interdisciplinary Patient‐Centred Care Intervention for People With Multimorbidity in Primary Care: A Qualitative Study,” BMJ Open 11 (2021): e046914, 10.1136/bmjopen-2020-046914. PMC8475135 34561255

[63] K. Fisher , M. Markle‐Reid , J. Ploeg , et al., “Self‐Management Program Versus Usual Care for Community‐Dwelling Older Adults With Multimorbidity: A Pragmatic Randomized Controlled Trial in Ontario, Canada,” Journal of Comorbidity 10 (2020): 96339, 10.1177/2235042X20963390. PMC7573753 33117723

[64] M. Fortin , M. Stewart , P. Ngangue , et al., “Scaling Up Patient‐Centered Interdisciplinary Care for Multimorbidity: A Pragmatic Mixed‐Methods Randomized Controlled Trial,” Annals of Family Medicine 19 (2021): 126–134, 10.1370/afm.2650. 33685874 PMC7939717

[65] L. O. Toole , D. Connolly , and S. Smith , “Impact of an Occupation‐Based Self‐Management Programme on Chronic Disease Management,” Australian Occupational Therapy Journal 60 (2013): 30–38, 10.1111/1440-1630.12008. 23414187

[66] A. Ryan , S. M. Smith , V. Cummins , C. Murphy , and R. Galvin , “Development and Feasibility of an Inter‐Agency Physical Activity and Education Programme for Adults With Multimorbidity in Primary Care: Activ8,” Journal of Multimorbidity and Comorbidity 12 (2022): 42350, 10.1177/26335565221142350. PMC9743022 36518523

[67] S. T. Skou , R. H. Brødsgaard , M. Nyberg , et al., “Personalised Exercise Therapy and Self‐Management Support for People With Multimorbidity: Feasibility of the MOBILIZE Intervention,” Pilot and Feasibility Studies 9 (2023): 12, 10.1186/s40814-023-01242-0. 36653858 PMC9847074

[68] T. Okpako , A. Woodward , K. Walters , et al., “Effectiveness of Self‐Management Interventions for Long‐Term Conditions in People Experiencing Socio‐Economic Deprivation in High‐Income Countries: A Systematic Review and Meta‐Analysis,” Journal of Public Health 45 (2023): 970–1041, 10.1093/pubmed/fdad145. 37553102 PMC10687879

[69] K. K. Frantzen and M. D. Fetters , “Meta‐Integration for Synthesizing Data in a Systematic Mixed Studies Review: Insights From Research on Autism Spectrum Disorder,” Quality & Quantity 50 (2016): 2251–2277, 10.1007/s11135-015-0261-6.

[70] M. B. Søgaard , K. Andresen , and M. Kristiansen , “Systematic Review of Patient‐Engagement Interventions: Potentials for Enhancing Person‐Centred Care for Older Patients With Multimorbidity,” BMJ Open 11 (2021): e048558, 10.1136/bmjopen-2020-048558. PMC8679112 34916309

[71] L. Steell , S. J. Krauth , S. Ahmed , et al., “Multimorbidity Clusters and Their Associations With Health‐Related Quality of Life in Two UK Cohorts,” BMC Medicine 23, no. 1 (2025): 1, 10.1186/s12916-024-03811-3. 39773733 PMC11708164

[72] Diabetes Prevention Program Research Group , “HbA1c as a Predictor of Diabetes and as an Outcome in the Diabetes Prevention Program: A Randomized Clinical Trial,” Diabetes Care 38 (2015): 51–58, 10.2337/dc14-0886. 25336746 PMC4274777

[73] K. Asmat , E. Sivarajan Froelicher , K. A. Dhamani , R. Gul , and N. Khan , “Effect of Patient‐Centered Self‐Management Intervention on Glycemic Control, Self‐Efficacy, and Self‐Care Behaviors in South Asian Adults With Type 2 Diabetes Mellitus: A Multicenter Randomized Controlled Trial,” Journal of Diabetes 16 (2024): e13611, 10.1111/1753-0407.13611. 39264007 PMC11391380

[74] T. Bilgehan and B. Vardar İnkaya , “The Effect of a Holistic Nurse Coaching Intervention on Glycemic Control, Diabetes Self‐Management, and Empowerment: A Randomized Controlled Trial,” BMC Nursing 24 (2025): 627, 10.1186/s12912-025-03252-0. 40457333 PMC12131424

[75] A. Barbato , B. D'Avanzo , M. Cinquini , et al., “Effects of Goal‐Oriented Care for Adults With Multimorbidity: A Systematic Review and Meta‐Analysis,” Journal of Evaluation in Clinical Practice 28 (2022): 371–381, 10.1111/jep.13674. 35355381 PMC9314986

[76] Y. Zhang , P. Alonso‐Coello , G. H. Guyatt , et al., “GRADE Guidelines: 19. Assessing the Certainty of Evidence in the Importance of Outcomes or Values and Preferences—Risk of Bias and Indirectness,” Journal of Clinical Epidemiology 111 (2019): 94–104, 10.1016/j.jclinepi.2018.01.013. 29452223

[77] EClinicalMedicine , “Social Prescribing: Addressing Societies Holistic Health‐Care Needs,” eClinicalMedicine 42 (2021): 101243, 10.1016/j.eclinm.2021.101243. 36313957 PMC9614554

[78] S. M. Smith , E. Wallace , C. Salisbury , M. Sasseville , E. Bayliss , and M. Fortin , “A Core Outcome Set for Multimorbidity Research (COSmm),” Annals of Family Medicine 16 (2018): 132–138, 10.1370/afm.2178. 29531104 PMC5847351

[79] J. Valabhji , E. Barron , A. Pratt , et al., “Prevalence of Multiple Long‐Term Conditions (Multimorbidity) in England: A Whole Population Study of Over 60 Million People,” Journal of the Royal Society of Medicine 117 (2023): 104–117, 10.1177/01410768231206033. 37905525 PMC11046366

[80] M. Alshakhs , B. Jackson , D. Ikponmwosa , R. Reynolds , and C. Madlock‐Brown , “Multimorbidity Patterns Across Race/Ethnicity as Stratified by Age and Obesity,” Scientific Reports 12 (2022): 9716, 10.1038/s41598-022-13733-w. 35690677 PMC9188579

[81] A. R. Quiñones , A. Botoseneanu , S. Markwardt , et al., “Racial/Ethnic Differences in Multimorbidity Development and Chronic Disease Accumulation for Middle‐Aged Adults,” PLoS One 14 (2019): e0218462, 10.1371/journal.pone.0218462. 31206556 PMC6576751

[82] B. Hayanga , M. Stafford , C. L. Saunders , and L. Bécares , “Ethnic Inequalities in Age‐Related Patterns of Multiple Long‐Term Conditions in England: Analysis of Primary Care and Nationally Representative Survey Data,” Sociology of Health & Illness 46 (2024): 582–607, 10.1111/1467-9566.13724. 37879907

[83] Z. Kayani , A. Willis , S. O. Salisu‐Olatunji , S. Jeffers , K. Khunti , and A. Routen , “Reporting and Representation of Underserved Groups in Intervention Studies for Patients With Multiple Long‐Term Conditions: A Systematic Review,” Journal of the Royal Society of Medicine 117 (2024): 302–317, 10.1177/01410768241233109. 38626808 PMC11529669

[84] K. Barnett , S. W. Mercer , M. Norbury , G. Watt , S. Wyke , and B. Guthrie , “Epidemiology of Multimorbidity and Implications for Health Care, Research, and Medical Education: A Cross‐Sectional Study,” Lancet 380 (2012): 37–43, 10.1016/S0140-6736(12)60240-2. 22579043

