# Supplementary material for: Effectiveness and Experiences of Self‐Management Interventions in Adults With Multiple Long‐Term Conditions: A Mixed Methods Systematic Review
Source: J Eval Clin Pract. 2026 Aug 20;32(5):e70565. doi: 10.1111/jep.70565 (PMC13492993; doi:10.1111/jep.70565)
Supplement: Supplementary file 1 [file JEP-32-0-s001.docx]

**S1 Figure. Search strategy for MEDLINE.**

Search strategy used for the database, MEDLINE, and was adapted for use across the other databases.

1	exp Multimorbidity/

2	exp Comorbidity/

3	exp Multiple Chronic Conditions/

4	multimorbid*.ti,ab.

5	multi-morbid*.ti,ab.

6	comorbid*.ti,ab.

7	co-morbid*.ti,ab.

8	("multiple long-term condition*" or "multiple long-term disease*" or "multiple long-term ill*").ti,ab.

9	("multiple chronic condition*" or "multiple chronic disease*" or "multiple chronic ill*").ti,ab.

10	(multidisease* or multi-disease*).ti,ab.

11	(multicondition* or multi-condition*).ti,ab.

12	1 or 2 or 3 or 4 or 5 or 6 or 7 or 8 or 9 or 10 or 11

13	exp Self-Management/

14	exp Self Care/

15	exp Self Efficacy/

16	exp Patient-Centered Care/

17	(patient focused or person focused).ti,ab.

18	(patient cent?red or person cent?red).ti,ab.

19	(patient orientated or person orientated).ti,ab.

20	patient activation.ti,ab.

21	self manag*.ti,ab.

22	self monitor*.ti,ab.

23	self care.ti,ab.

24	self efficacy.ti,ab.

25	13 or 14 or 15 or 16 or 17 or 18 or 19 or 20 or 21 or 22 or 23 or 24

26	12 and 25

27	exp animals/ not humans.sh.

28	26 not 27

29	limit 28 to english language

**S1 Table. Direction of effect plot for primary outcomes.**

Direction of effect plot using the vote counting method for quality of life and psychological outcomes, adapted from Boon and Thomson (2021).

| **Study ID** | **Study Design** | **Population (sample size)** | **Risk of bias** | **Quality of life** | **Mental health** | **Self-efficacy** |
| --- | --- | --- | --- | --- | --- | --- |
| **Boult2011** | Cluster RCT | 904 | High |  |  |  |
| **Contant2019** | RCT | 281 | Low |  |  |  |
| **Fisher2020** | RCT (mixed methods) | 59 | Low | ▼ | ▲ |  |
| **Fortin2021a** | RCT (mixed methods) | 284 | Low |  | ◄► |  |
| **Fortin2021b** | Pre-post | 284 | High | ◄► | ▲* | ▲ |
| **Garvey2015** | RCT | 50 | Low | ▲* | ▲ |  |
| **Hwang2025** | RCT | 42 | Low | ◄► | ▲* |  |
| **Khunti2021** | RCT | 353 | Unclear | ▼* | ◄► | ◄► |
| **Lear2021** | RCT | 230 | Low | ◄► |  |  |
| **Mercer2016** | RCT | 152 | Low | ▲ | ▲ | ▲ |
| **O'Toole2013** | Pre-post (mixed methods) | 16 | Low | ◄► | ◄► | ▲ |
| **O'Toole2021** | RCT | 149 | Unclear | ▲ | ▲ | ▲ |
| **Panagioti2018** | RCT within cohort | 1306 | Unclear | ▲ | ▲ |  |
| **Park2014** | RCT | 43 | High |  |  |  |
| **Pellet2024** | Pre-post | 138 | High |  |  |  |
| **Reed2018** | RCT | 254 | Low |  |  |  |
| **Ryan2022** | Pre-post (mixed methods) | 19 | Low | ▲ |  |  |
| **Skou2023** | Pre-post (mixed methods) | 20 | Low |  |  |  |
| **Skou2025** | RCT | 228 | Low | ▲* | ▲ | ▲ |
| **Sommers2000** | Controlled cohort study | 543 | High |  | ◄► |  |
| **Struwe2020** | RCT | 91 | High |  |  |  |
| **Williams-Livingston2020** | Cross-sectional | 93 | High |  |  |  |
| **Yang2022** | RCT | 136 | High | ▼ |  | ▲* |
| RCT: Randomised Controlled Trial  Effect direction: upward arrow ▲* = statistically significant positive health impact, upward arrow ▲= positive health impact, downward arrow ▼* = statistically significant negative health impact, downward arrow ▼= negative health impact, sideways arrow ◄►= no change/mixed effects observed | | | | | |  |

**S2 Table. Direction of effect plot for behavioural outcomes.**

Direction of effect plot using the vote counting method for behavioural outcomes, adapted from Boon and Thomson (2021).

| **Study ID** | **Study Design** | **Population (sample size)** | **Risk of bias** | **Self-management** |  | **Lifestyle changes** | **Ability to perform daily activities** | **Frequency of activity participation** | **Occupational performance** | **Social support** | **Goal setting** |
| --- | --- | --- | --- | --- | --- | --- | --- | --- | --- | --- | --- |
| **Boult2011** | Cluster RCT | 904 | High |  |  |  |  |  |  |  |  |
| **Contant2019** | RCT | 281 | Low | ▲* |  |  |  |  |  |  |  |
| **Fisher2020** | RCT (mixed methods) | 59 | Low |  |  |  |  |  |  |  |  |
| **Fortin2021a** | RCT (mixed methods) | 284 | Low | ◄► |  | ◄► |  |  |  |  |  |
| **Fortin2021b** | Pre-post | 284 | High | ◄► |  | ◄► |  |  |  |  |  |
| **Garvey2015** | RCT | 50 | Low | ◄► |  |  | ▲* | ▲* | ▲* |  | ▲* |
| **Hwang2025** | RCT | 42 | Low | ◄► |  |  |  |  |  |  |  |
| **Khunti2021** | RCT | 353 | Unclear |  |  | ▼ |  |  |  |  |  |
| **Lear2021** | RCT | 230 | Low | ▲ |  |  |  |  |  | ◄► |  |
| **Mercer2016** | RCT | 152 | Low |  |  |  |  |  |  |  |  |
| **O'Toole2013** | Pre-post (mixed methods) | 16 | Low |  |  |  | ▲ | ▲* | ▲* |  |  |
| **O'Toole2021** | RCT | 149 | Unclear |  |  |  | ▲ | ▲ | ▲ |  |  |
| **Panagioti2018** | RCT within cohort | 1306 | Unclear | ◄► |  |  |  |  |  |  |  |
| **Park2014** | RCT | 43 | High | ▲* |  |  |  |  |  |  | ◄► |
| **Pellet2024** | Pre-post | 138 | High | ▲* |  |  |  |  |  |  |  |
| **Reed2018** | RCT | 254 | Low | ◄► |  | ▲ |  |  |  |  |  |
| **Ryan2022** | Pre-post (mixed methods) | 19 | Low |  |  |  |  |  |  |  |  |
| **Skou2023** | Pre-post (mixed methods) | 20 | Low |  |  |  |  |  |  |  |  |
| **Skou2025** | RCT | 228 | Low |  |  |  |  |  |  |  |  |
| **Sommers2000** | Controlled cohort study | 543 | High |  |  | ◄► |  |  |  | ◄► |  |
| **Struwe2020** | RCT | 91 | High |  |  |  |  |  |  |  |  |
| **Williams-Livingston2020** | Cross-sectional | 93 | High |  |  |  |  |  |  |  |  |
| **Yang2022** | RCT | 136 | High | ◄► |  |  |  |  |  | ◄► |  |
| RCT: Randomised Controlled Trial  Effect direction: upward arrow ▲* = statistically significant positive health impact, upward arrow ▲= positive health impact, downward arrow ▼* = statistically significant negative health impact, downward arrow ▼= negative health impact, sideways arrow ◄►= no change/mixed effects observed | | | | | | | | | | | |

**S3 Table. Direction of effect plot for clinical outcomes.**

Direction of effect plot using the vote counting method for clinical outcomes, adapted from Boon and Thomson (2021).

| **Study ID** | **Study Design** | **Population (sample size)** | **Risk of bias** | **Health status** | **Clinical measures** | **Adherence** | **Physical functioning** | **Treatment** |
| --- | --- | --- | --- | --- | --- | --- | --- | --- |
| **Boult2011** | Cluster RCT | 904 | High |  |  |  |  |  |
| **Contant2019** | RCT | 281 | Low |  |  |  |  |  |
| **Fisher2020** | RCT (mixed methods) | 59 | Low |  |  |  |  |  |
| **Fortin2021a** | RCT (mixed methods) | 284 | Low | ▼ |  |  |  |  |
| **Fortin2021b** | Pre-post | 284 | High | ▲* |  |  |  |  |
| **Garvey2015** | RCT | 50 | Low |  |  |  |  |  |
| **Hwang2025** | RCT | 42 | Low | ◄► | ◄► | ▲* |  |  |
| **Khunti2021** | RCT | 353 | Unclear |  | ◄► | ◄► |  |  |
| **Lear2021** | RCT | 230 | Low |  |  |  |  |  |
| **Mercer2016** | RCT | 152 | Low |  |  |  |  |  |
| **O'Toole2013** | Pre-post (mixed methods) | 16 | Low |  |  |  |  |  |
| **O'Toole2021** | RCT | 149 | Unclear |  |  |  |  |  |
| **Panagioti2018** | RCT within cohort | 1306 | Unclear |  |  |  |  |  |
| **Park2014** | RCT | 43 | High | ◄► |  |  |  |  |
| **Pellet2024** | Pre-post | 138 | High |  |  |  |  |  |
| **Reed2018** | RCT | 254 | Low | ◄► |  |  |  |  |
| **Ryan2022** | Pre-post (mixed methods) | 19 | Low |  | ▲ |  |  |  |
| **Skou2023** | Pre-post (mixed methods) | 20 | Low |  |  |  |  |  |
| **Skou2025** | RCT | 228 | Low |  |  |  | ▲ |  |
| **Sommers2000** | Controlled cohort study | 543 | High | ◄► | ◄► |  | ◄► |  |
| **Struwe2020** | RCT | 91 | High |  |  |  |  |  |
| **Williams-Livingston2020** | Cross-sectional | 93 | High |  |  |  |  |  |
| **Yang2022** | RCT | 136 | High |  |  | ▲ |  | ◄► |
| RCT: Randomised Controlled Trial  Effect direction: upward arrow ▲* = statistically significant positive health impact, upward arrow ▲= positive health impact, downward arrow ▼* = statistically significant negative health impact, downward arrow ▼= negative health impact, sideways arrow ◄►= no change/mixed effects observed | | | | | | | | |

**S4 Table. Direction of effect plot for health service outcomes.**

Direction of effect plot using the vote counting method for health service outcomes, adapted from Boon and Thomson (2021).

| **Study ID** | **Study Design** | **Population (sample size)** | **Risk of bias** | **Health service use** |
| --- | --- | --- | --- | --- |
| **Boult2011** | Cluster RCT | 904 | High | ◄► |
| **Contant2019** | RCT | 281 | Low |  |
| **Fisher2020** | RCT (mixed methods) | 59 | Low | ▲ |
| **Fortin2021a** | RCT (mixed methods) | 284 | Low |  |
| **Fortin2021b** | Pre-post | 284 | High |  |
| **Garvey2015** | RCT | 50 | Low | ◄► |
| **Hwang2025** | RCT | 42 | Low |  |
| **Khunti2021** | RCT | 353 | Unclear |  |
| **Lear2021** | RCT | 230 | Low | ▲ |
| **Mercer2016** | RCT | 152 | Low |  |
| **O'Toole2013** | Pre-post (mixed methods) | 16 | Low |  |
| **O'Toole2021** | RCT | 149 | Unclear | ▲ |
| **Panagioti2018** | RCT within cohort | 1306 | Unclear |  |
| **Park2014** | RCT | 43 | High |  |
| **Pellet2024** | Pre-post | 138 | High | ◄► |
| **Reed2018** | RCT | 254 | Low | ▼ |
| **Ryan2022** | Pre-post (mixed methods) | 19 | Low |  |
| **Skou2023** | Pre-post (mixed methods) | 20 | Low |  |
| **Skou2025** | RCT | 228 | Low |  |
| **Sommers2000** | Controlled cohort study | 543 | High | ▲ |
| **Struwe2020** | RCT | 91 | High | ◄► |
| **Williams-Livingston2020** | Cross-sectional | 93 | High |  |
| **Yang2022** | RCT | 136 | High | ◄► |
| RCT: Randomised Controlled Trial  Effect direction: upward arrow ▲* = statistically significant positive health impact, upward arrow ▲= positive health impact, downward arrow ▼* = statistically significant negative health impact, downward arrow ▼= negative health impact, sideways arrow ◄►= no change/mixed effects observed | | | | |

**S5 Table: Summary of findings from thematic synthesis**

Summary of qualitative findings from thematic synthesis, including analytical themes, descriptive themes, codes and studies contributing to the finding,

| **Analytical theme** | **Descriptive themes** | **Example quotes** | **Codes** | **Studies contributing to finding** |
| --- | --- | --- | --- | --- |
| ***Interdisciplinary collaboration can facilitate better support*** | Benefits of interdisciplinary collaboration as part of support | “*helpful because we discuss the client in detail, with enthusiasm, and from a social point of view as well. So, I found that was very helpful to help you approach the client*" (Health care professional; Fisher *et al.*, 2020) | Interdisciplinary approach facilitated the intervention; Interdisciplinary collaboration improved cohesion in the care of patients; Interdisciplinary collaboration improved team work and socialisation: interdisciplinary working improved trust between disciplines; interdisciplinary working was effective and maintained autonomy of HCPs; intervention team meetings allowed in depth discussion of patients; team collaboration improved communication between disciplines | Ryan et al., (2022); Fisher et al., (2020); Ngangue et al., (2020) |
|  | Communication between providers facilitated delivery of support | *‘Since the implementation of the intervention, we take 5 minutes to discuss, either at the end or before dinner [the day] … that allows us to ensure better follow- up’* (Nurse; Ngangue *et al.*, 2021) | Appreciated interdisciplinary communication involved in care planning; communication between HCPs helped patient follow up; communication facilitated interdisciplinary collaboration; communication helped maintain HCPs collaboration and implementation; face to face communication between HCPs was important; HCPs on same site led to easier communication and collaboration across disciplines; indirect and direct communication important for interdisciplinary approach; indirect communication facilitated interdisciplinary communication; regular interdisciplinary meetings helped implementation; regular team meetings allowed opportunities to share knowledge; team meetings helped health providers understand the role of different disciplines | Fisher et al., (2020); Ngangue et al., (2020); Ngangue et al., (2021) |
|  | Communication facilitated support and care | *“Ensure that there are people in place to support it, that people feel comfortable doing it, have the proper training, and that there are also interdisciplinary relationships between nurses and other health professionals. Make sure that they understand what the intervention consists of and how to intervene with this clientele”* (Practice manager; Ngangue *et al.*, 2021) | Communication important to implement intervention; communication regarding intervention was important for implementation; feed information to participants slowly; help participants understand health appointments; supported patients with visits to health appointments | Ngangue et al., (2021); Doyle et al. (2022) |
|  | Communication, collaboration and support needed to set goals | *“HCPs highlighted the importance of discussions with the patient to set realistic and appropriate goals to preserve their motivation and make positive changes towards their health behaviours.”* (Author quote; Ngangue *et al.*, 2020) | Collaboration between HCPs and patients to identify goals; collaboration between patients and HCPs for care decisions; conversations between patients and HCPs were important to set realistic goals; HCPs respected patients’ needs when setting goals | Doyle et al. (2022); Ngangue et al., (2020) |
|  | Providers valued input from other disciplines to help deliver support and care | *“We have the PSW (personal support worker) coming in the meeting as well, and I really liked that .. . during those meetings, they actually have a say and express what they think about the client and also the caregiver."* (Health care professional; Fisher *et al.*, 2020) | Importance of including HCPs from other disciplines in care planning; interdisciplinary team meetings allowed for input from other HCPs; new additions to care team were important; personal support worker was more knowledgeable about patient; valuable information provided from HCPs across different disciplines | Fisher et al., (2020) |
| ***Influential external and contextual factors*** | External and contextual factors influence health, management and engagement with support | "*It’s alright saying ‘go and do these things’ but if you’ve not got the money to go a.. .’cause like I can get eh the Glasgow thing, and you get for half price, but even half price I cannae afford that either because eh finances.*” (Patient; McCallum *et al.*, 2021) | Account for contextual factors when developing interventions; Anger and frustration at lack of autonomy due to external factors (lack of finances, facilities); Anger at having to pay taxes and feeling sick pay threatened; Contextual issues limited participation in sessions; Importance of understanding patient context for successful self-management; Low engagement with signposted services due to external factors; Personal and social circumstances were barriers to self-management; Work commitments limits time to exercise or attend sessions | Khan et al., (2018); McCallum et al., (2021); Skou et al. (2023) |
|  | External factors impacting delivery of support | *"The health system reform was undoubtedly a difficulty because this great project happened simultaneously as the whole reorganization"* (Practice manager; Ngangue *et al.*, 2021) | External factors affected the communication between HCPs regarding patient follow up; Health system reorganisation affected communication across disciplines; Health system reorganisation influenced implementation; Intervention was difficult to deploy due to national health system reorganisation; Reorganisation of health system presented difficulties with implementing intervention | Ngangue et al., (2021) |
|  | Feeling isolated from support providers | *“The problem is I don't know what the picture [refers to uploading photos of meals consumed to better track their diet to manage their diabetes] is doing. Like there's no one analyzing the picture—is there?"* (Patient; Khan *et al.*, 2018) | Limited feedback left patients feeling isolated and discouraged; Poor relationship with GP and social isolation had negative impact on wellbeing; Tool users feel isolated and discouraged due to lack of feedback from health providers | Khan et al., (2018); McCallum et al., (2021) |
|  | Feelings that support will not help their situation | *"To be honest, as I said, I – I just – I suppose it – If there was something they could do for me then yes, it would be, but I just feel there’s nothing, you know, out there for them to give me, if you like."* (Patient; McCallum *et al.*, 2021) | Felt that signposted services would not help; Felt that there was no solution to problems due to symptoms and social context; Participants felt that solutions were not useful due to personal or social circumstances; Participants felt that there was nothing that could be done; Participants had a sense of hopelessness; Sense of reluctance to try new things | McCallum et al., (2021) |
|  | Impact of contextual factors on support and self-management | “*I do not get [the home-exercise] done. Well, there is so much else I need to do. Well, I have a house and a garden, and it must be kept, and – yes.”*  (Participant: Skou *et al.*, 2023) | HCPs could easily identify barriers to self-management; HCPs could easily identify motivations and barriers; HCPs improved understanding of patient issues and how this affected self-management; Intervention allowed for early identification and prevention; Lack time or motivation to perform exercises; Tool offered health providers insights into contextual factors that inhibit self-management | Khan et al., (2018); Ngangue et al., (2021); Skou et al. (2023) |
|  | Mental health issues affected engagement with support activities | *"I started going back. But I stopped going recently because of all this greeting (crying) again. I’m, no going to sit in a club and started greeting right."* (Patient; McCallum *et al.*, 2021) | Mental health problems were prevalent; Poor mental health was a barrier to engaging in social activities | McCallum et al., (2021) |
|  | Negative impact of multimorbidity on health and management | *"When [the patient] was experiencing a lot of pain...the other goals drop off because she's not going to be attempting those."* (Health care professional, Khan *et al.*, 2018) | Complexity of multimorbidity impacts HCPs decision re. self-management; HCPs reported challenges multimorbidity presents for self-management; Medical and social complexity in multimorbidity; Negative impact of multimorbidity on daily life and sense of competence; Negative Impact of symptoms on achieving goals; Nurses admired patients outlook and engagement; Patients are complicated, medically, socially and psychologically; Physical and mental symptoms affected daily life; Symptoms of conditions limited participation | Khan et al., (2018); McCallum et al., (2021); Doyle et al. (2022); Skou et al. (2023) |
|  | Patients had other priorities beyond health | “*They were not only caring for themselves but also for their spouses, supporting them to navigate the system and manage their conditions”*  (Author quote, Skou *et al.*, 2023) | Caring responsibilities meant that own health was not prioritised; Priorities were social issues, not health | McCallum et al., (2021); Skou et al. (2023) |
|  | Self-management is a long-term and multilayered process | *"What I usually do with people is just layers and layers and layers and layers of therapy...And you can't even get to the therapy because... they're at risk for homelessness. Then they're not getting their Ontario Works cheque. And so you're just kind of going along and along. It takes me a long time to meet with somebody once you get through all of that stuff and then go, “Let's set some goals."* (Health care professional; Khan *et al.*, 2018) | Self-management is long-term and multilayered process | Khan et al., (2018) |
|  | Effective communication needed to maintain therapeutic relationship | *‘Well, listening to what I had to say… like eating fruits, it is difficult for me. She [the nutritionist] was listening… she took into account what I said’* (Patient; Ngangue *et al.*, 2020) | Active listening by HCPs to make good care decisions; Communication between practitioners and patients was important; HCPs engaged in active listening during intervention consultations; Nurses used various techniques to build rapport with patients; Patient appreciated HCPs efforts to help them understand more complex ideas; Patients appreciated the active listening of HCPs; Patients' spouse appreciated the encouragement from HCPs | Doyle et al. (2022), Ryan et al., (2022); Ngangue et al., (2020) |
|  | Effective support improved therapeutic relationship | "*The consultation was more relaxed. I actually felt I got to know the doctor a bit better as well because I was managing to spend more time with him. Sometimes you just go in, tell him your problems, examine you and he says ‘that’s what’s wrong’. But it wasnae like that. I got to.. ..He made me feel more relaxed for some reason. I don’t know why*." (Participant; McCallum *et al.*, 2021) | Improved relationship with GP; Increased confidence with communicating with GP | McCallum et al., (2021); O’Toole et al., (2013) |
|  | Group support encouraged socialisation and peer support | "*Listening to other people you know you’re not alone. Other people are the same*" (Participant; O'Toole *et al.*, 2013) | Diverse conditions but share similar problems; Group based nature of intervention was beneficial; Group discussions encouraged participations and problem-solving; Group interactions encouraged attendance; Group interactions reduced feelings of isolation; Group programme gave participants a chance to meet new people; Group programme improved socialisation; Group session were enjoyable and provided motivation for participants; Group sessions enabled peer learning; Group sessions for goal setting and sharing successes was motivational; Group setting allowed for socialisation; Group setting reduced feeling of isolation; Participants enjoyed meeting new people through group sessions; Sense of security felt in a group intervention and participants felt comfortable sharing experiences | O’Toole et al., (2013); Ryan et al., (2022); Skou et al. (2023) |
|  | Importance of peers providing support | *“Other peoples’ problems seem much worse than mine and they still manage, now I say ‘well they’re keeping going why can’t I keep going myself?’ and basically that’s how it’s helped me*" (Participants; O'Toole *et al.*, 2013) | Improved confidence in group peer support; Interaction with peers provided motivation and goal setting; Learning from peers during group sessions; Participants felt more open and comfortable at the group intervention; Participants observed others coping well, despite many difficulties, during group sessions; Peer support and comparison with others' situations provided motivation; Peer support provided sense of control over life and health; Peer support was important; Support and supervised sessions allowed for feelings of safety | Ryan et al., (2022); O’Toole et al., (2013); McCallum et al., (2021); Skou et al. (2023) |
|  | Importance of relationships with social and family networks for effective support | *“As the interviews focused on participants’ experiences of CARE Plus, much of the accounts of relatedness focused on the participant’s relationships with their named practitioner. Nevertheless, all participants in the major/moderate improvement group also described at least one other important relationship, speciﬁcally with a family member or social care professional*" (Author quote; McCallum *et al.*, 2021) | Education and communication with family and carers; Family and carers helped support patient's health; Family and informal carers important role in motivation; HCPs built relationship with family and carers of patients; Importance of relationships between family, friends and health professionals; Importance of social networks and activities on self-management behaviours; Nurses advised carers and patients to take them to hospital or HCPs; Pre-existing support network improved physical activity | Doyle et al. (2022); McCallum et al., (2021); |
|  | Poor social and family support network impacted support received | *"People say to me ‘god I know how you feel I’m on my own myself’. I says ‘Are you? Did your grandson stay last night?’ ‘Aye’ ‘Did your daughter came over to visit you this morning?’ ‘Aye’. ‘You’re not on your own I’m here 24/7, the door doesnae go, the phone doesnae go, nothing. So I wake up ‘what’s the point of getting up? What’s the point of cleaning up?’ Right so you get up.. . och I go back to my bed"* (Patient; McCallum *et al*., 2021) | Existing family support was limited with unsupportive and obstructive relationships; Socially isolated and had problematic relationships with family, friends | McCallum et al., (2021) |
|  | Positive therapeutic relationship important for effective support | *“But they would still ask for that speciﬁc person with whom they had the most contact”*  (Nurse; Doyle *et al.*, 2022) | Body language of HCPs important to maintain therapeutic relationships with patients; Build trust with patients to help make care easier; Emotional and social support from nurses; Participants appreciated and valued relationship with GP; Positive relationship with nurses; Pre-existing positive relationship with health professionals; Reported good relationships with GPs; Support from nurses was reassuring for patients | Doyle et al. (2022), Ngangue et al., (2020); McCallum et al., (2021) |
|  | Therapeutic relationship posed challenges in delivery of support | "*she was ‘overly-friendly’ with her GP, which stopped her conﬁding difﬁcult things. [they] know me too well – what is, in a way, sometimes I think I maybe walk up and I go in and then don’t end up telling them what I’m there for.. .. me and the doctor end up, laughing about something and I actually – and then I think, ‘No, I don’t wanna talk about what I came here for’, which I know sounds really strange*" (Participant; McCallum *et al.*, 2021) | Did not want to discuss broader issues beyond medical problems with GPs; Found intervention consultations awkward; Participant described 'over friendly' relationship with GP which stopped confiding difficult issues; Participants described dissatisfaction with relationship with GP; Pre-existing poor relationship with GP; Pre-existing relationship with GP prevented being honest about problems | McCallum et al., (2021) |
|  | Valued consultations with support providers | "*All these meetings made me see what my health status was. Also, at the same time, I was reassured in the sense that if I had other health problems. I would be able to find care that I need, know where to look for my care…*" (Participant; Ngangue *et al.*, 2020) | Able to discuss broader issues at GP consultations; Consultations reassured patients about their conditions and care; Longer consultations allowed more time to talk about problems; More time to listen and build relationships with patients; More time to react to patient issues; More time with patients facilitated holistic care; Participants reported receiving longer consultations; Participants valued longer consultations with GPs; Support provided nurses with more time to care; Valued the chance to be listened to with extra time and opportunity to discuss broader issues at GP intervention consultations | McCallum et al., (2021); Ngangue et al., (2020); Doyle et al. (2022) |
| ***Importance of individualisation and empowerment*** | Active role of patient in their own care | *When I visit now, when I am speaking to the patients, umm, I try and look at it from their point of view, and let them direct where things are going. Instead of dictation, because it’s in us to take over right? So, you know, allowing the patients to kind of lead where things are going and set the goals.*” (Health providers; Fisher *et al.*, 2020) | HCPs allowed patients to direct consultations and set goals; patient centred approach of intervention focused on emotions and care of the patient; patient centred care approach allowed for active patient involvement in their own care | Fisher et al., (2020); Ngangue et al., (2020) |
|  | Autonomy is important for those receiving support | *One participant in the slight change group did demonstrate a degree of autonomy: she had attended a local mental health group (after signposting from her GP) and had referred herself to a counselling service.* (Author quote; McCallum *et al.*, 2021) | Autonomy of participants was important; lack of autonomy due to finances and lack of facilities; lack of autonomy negatively impacted wellbeing and was a barrier to future change; participants demonstrated autonomy | McCallum et al., (2021) |
|  | Feel empowered by support received | *“The emphasis is just on movement and it’s in an environment that a lot of them are like ‘oh my goodness I, I actually came to a leisure centre and I was on a bike or’ so it’s really empowering for them"* (Health provider; Ryan *et al.*, 2022) | Environment and group setting empowered participants; intervention approach allowed empowerment of participants; values strength-based approach as it empowers patients | Ryan et al., (2022); Fisher et al., (2020) |
|  | Important to maintain autonomy of support providers | *‘I have a good opinion of it because it puts the overall competence of the nurse in the foreground, both at the evaluative level, the teaching level and at the collaborative level. It positions nursing … in its pivotal role’ (*Practice manager; Ngangue *et al.*, 2021) | Autonomy of practitioners was important for inter-disciplinary relationships; emphasises the important role of HCPs (nurses); intervention enabled HCPs to focus on their roles and skills to manage patients | Ryan et al, (2022); Ngangue et al., (2021) |
|  | Need to individualise and adapt support depending on needs and priorities | *"Not everybody needs the same questions. Everybody's case is different...but we got the exact same questions." (*Patient; Khan *et al.*, 2018) | Adapt intervention components and timings to suit patients; adapt self-regulation activities and monitoring based on patient; adapt to changing self-management needs in patients with multimorbidity; expand tools capabilities to account for complexity of multimorbidity; greater personalisation and customisability of goals needed; HCPs adapted intervention to reflect patient context and reality; HCPs appreciated personalised interventions; HCPs need to meet the changing needs of patients; holistic care needed for people with MLTC; keep patient needs and priorities at the centre of care and decisions; lack of personalisation of the self-management tool; need to adapt intervention based on participant context; need to personalise the intervention around the patient; patients appreciated the holistic, patient centred approach of the intervention; support programmes provided holistic assessment and care of patient | O’Toole et al., (2013); Khan et al., (2018); Ngangue et al., (2020); Doyle et al. (2022); Skou et al. (2023) |
|  | Realistic and achievable goals important as part of support | *‘We often tell patients that a little change, even better than a big change because we gradually develop the lifestyle habit they do not have… with more realistic goals’.* (Nurse; Ngangue *et al.*, 2020) | Adapting goals to patients needs was important for permanent change; Goals based on observable outcomes; Goals set were often related to physical health; Goals set were related to leisure and productive activities; HCPs focused on clear, realistic objectives; HCPs set individualised, realistic goals for patients - therapeutic relationship; Need to set realistic, achievable goals to maintain motivation; Realistic goals offered potential for permanent changes | Ngangue et al., (2020); Khan et al., (2018); O’Toole et al., (2013) |
|  | Realistic goal setting improved confidence and motivation | *"As long as you’ve achieved something you’re not going to disachieve it. You want to keep going and build on that conﬁdence and move on "* (Participant; O'Toole *et al.*, 2013) | Goal setting improved feelings of competence; Observable goals were motivational for participants; Realistic goals maintained motivation and encouraged positive behaviours; Successful achievement of goals for participants provided motivation and confidence | McCallum et al., (2021); Ngangue et al., (2020); O’Toole et al., (2013) |
|  | Recommendations for greater individualisation of support delivery | *"I think the bottom line that comes out of my disconcertedness about it was that the questions were too broad. They weren't specific enough."* (Patient; Khan *et al.*, 2018) | Electronic tool needed to be more specific to patients needs; Expand scope of intervention to reflect individual context; Flexible approach to self-management needed; Lack personalisation to account for individual context | Khan et al., (2018) |
|  | Support enabled people to take control over their own health | *Now, I find that he takes charge of his health, so that (the interventions) might have made a difference… it is good for sure’* (Caregivers; Ngangue *et al.*, 2020) | Behaviours and health within their own control; Greater control over own health; HCPs provided education to encourage them to take responsibility for health; HCPs role in educating and motivating participants; Patients' caregiver reported that the intervention had encouraged patient to take control of their health; Prioritise themselves and their health over anything else | McCallum et al., (2021); Fortin et al., (2021a); Ngangue et al., (2020); Doyle et al. (2022) |
| ***‘Real world’ implementation*** | Complex delivery of support posed challenges | "*We did not have any document to give to patients, to explain what [the intervention] meant. It was difficult for us to explain it*" (Providers; Ngangue *et al.*, 2021) | Barrier to implementation - complexity of intervention; Difficult to explain intervention concepts to patients; Health providers found the intervention complex, with difficult concepts; Needed help to clearly explain the intervention to participants | Ngangue et al., (2021) |
|  | Factors impacting interdisciplinary collaboration | *‘The collaboration with the doctors was not a success. I was afraid to refer clients’* (Health care professionals, Ngangue *et al.*, 2021) | Fear of referring patients to HCPs in other disciplines; Part-time schedules for HCPs was a barrier for communication across disciplines | Ngangue et al., (2021); Ngangue et al., (2020) |
|  | Factors impacting setting goals as part of support | *But it was very difficult for him...he found having to look at that information depressing...all of which points to the fact that he was not ready to set those goals.*" (Health care professionals; Khan *et al.*, 2018) | Difficult to set goals due to overwhelming, depressing information | Khan et al., (2018) |
|  | Issues with delivery of support | ‘*The only problem I have… is that calls are not answered… I find it very deplorable in the system…*’ (Caregiver; Ngangue *et al.*, 2020) | Fear of being referred to hospital; Intervention tool not suitable for all patients; Issues with information overload when communicating with patients; Issues with offering social support directly via the electronic tool; Issues with patient follow up due to health care absences; Issues with services provided during intervention; Monitoring calls from nurses caused anxiety for patients; Nurses calls and advice were inconvenient and occurred too often; Small number of study participants; Used the digital platform less often as time progressed | Khan et al., (2018); Ngangue et al., (2020); Fisher et al., (2020); Doyle et al. (2022) |
|  | Differences between information provided from support programme and provided from regular healthcare team | *“[they called] about my pulse. That it was too low. But that does not matter that it is too low. The cardiologist knows and he is not worried”*  (Participant, Doyle *et al.*, 2022) | Differences between what the support nurses said and what the regular healthcare team thought; Education provided filled gap left by regular GP appointments; Patients discussed their use of the digital platform with their regular healthcare team | Doyle et al. (2022) |
|  | Limitations to delivery of support | "*they felt the tool had limitations in terms of the comprehensiveness of its scope*" (Author quote; Khan *et al.*, 2018) | Intervention experience was similar to normal experience; Limitations in comprehensiveness of intervention | McCallum et al., (2021); Khan et al., (2018) |
|  | Limited progress towards support goals impacted motivation | "*A lot of times when people set goals, it doesn't work, especially the first time or couple of times...A lot of people set goals that are way too big, way too out there, or not realistic until they actually apply it...So then that sets them up for dropping out maybe. Because they're like, oh, I can't do this goal, I failed, I suck."* (Health care professional; Khan *et al.*, 2018) | Limited progress towards goals impacted motivation and confidence; Progress towards goals fluctuated throughout the day; Self-tracking goals impacted motivation and mental wellbeing; Unrealistic goals and limited progress negatively impacted motivation of participants | Khan et al., (2018) |
|  | No benefit from the support received | ‘*I don’t think I, I don’t think I’d use it, myself, I feel it’s a bit of a waste of time… cause the things you’re doing you can do by walking or y’know*’ (Participant; Ryan *et al.*, 2022) | Intervention not seen as beneficial; No improvements reported post-intervention; Physical activity intervention not seen as useful; Showed little or no motivational change | McCallum et al., (2021); Ryan et al., (2022) |
|  | Providers did not consistently adhere to delivery protocol | *Family physicians do not adhere to the [intervention] philosophy. This approach consists of identifying client needs. They still tend to say: ‘I am the doctor, I know what you need. I will tell you what you need, then apply it’* (Practice manager; Ngangue *et al.*, 2021) | HCPs did not adhere to intervention principles; HCPs returned to using familiar and efficient methods; Intervention self-management pack not received or not used by participants; Lack of appropriate collaboration among HCPs; Low participation rates by family physicians; Poor attendance at inter-disciplinary team meetings | Ngangue et al., (2021); McCallum et al., (2021); Fisher et al., (2020) |
|  | Providers lacked resources needed for delivery of support | *One constraint was the lack of physical space in some FMGs. Therefore, some healthcare professionals could not meet together to discuss patient action plans*’ (Practice manager; Ngangue *et al.*, 2021) | Lack of available premises and health professionals; Lack of infrastructure and resources negatively impacted interdisciplinary collaboration; Lack of infrastructure for team meetings | Ngangue et al., (2021) |
|  | Providers lacked time for appropriate discussion and support | *‘This is a problem because I only work a half-day per week at the clinic. Sometimes, patients do not need to see me during the time I am there. Also, when I am there, I have my schedule, so I may not have time to do something else. Then, the ideal option would be to always be there"* (Health care professional; Ngangue *et al.*, 2020) | HCPs lacked enough time to see patients; HCPs not have enough time to discuss patients with colleagues; Irregular consultations with GP throughout intervention; Lack of availability of HCPs; Lack of time available for HCPs to see patients; Part-time schedule of HCPs posed challenges to interdisciplinary collaboration | Ngangue et al., (2020); Ngangue et al., (2021); McCallum et al., (2021) |
|  | Staff turnover was an issue with providing and delivery of support | *‘There has been much movement with nurses during the last two years… for patients; it can be disruptive. If we had nurses that were well integrated with a continuum and were also able to do the link between the different HCPs to make sure there has follow-ups, coordination, all this’*. (Health care professional; Ngangue *et al.*, 2020) | Staff turnover posed challenges to team collaboration; Turnover of nurses caused problems with follow up | Ngangue et al., (2020) |
|  | Support provided did not fit with current practice | *Several providers revealed that the ePRO tool did not match existing approaches to goal setting used in the primary care practice. (*Author quote; Khan *et al.*, 2018) | Intervention principles were not compatible with philosophy and practice; Process for goal setting did not fit with existing framework used by health providers | Ngangue et al., (2021); Khan et al., (2018) |
|  | Appreciation for approaches to deliver support | *‘I thought the fact that it was so broad, the fact it was multi, so, anything from depression to diabetes to that was very practical to the cohort of people that I see every day… Yet it meant that, you could easily ﬁnd these people, y’know this covers a lots of people’* (Facilitator; Ryan *et al.*, 2022*)* | Broad focus of intervention (multimorbidity) allows for greater generalisability; Clear explanations of intervention components were appreciated by HCPs; HCPs appreciated patient centred and motivational approach; HCPs appreciated patient-centred approach; HCPs appreciated the preventative nature of the intervention; Patient appreciates the multidisciplinary distribution of care across health providers; Patient centred approach used effectively by HCPs; Patients acknowledged the tool's potential to enable self-management support from multiple disciplines | Ryan et al., (2022); Ngangue et al., (2020); Ngangue et al., (2021); Khan et al., (2018) |
|  | Coordination and finances needed to deliver support effectively | *Receiving funds to help us get started is always welcome rather than reorganizing activities using our resources’* (Practice manager; Ngangue *et al.*, 2021) | Administrative staff important to coordinate and monitor patient appointments; Administrative support was important for implementation; Administrative support for coordinating appointments was beneficial; Financial support needed for implementation; Leadership important for coordinating the intervention; Participants valued supervision from the facilitators; Supervisory team to coordinate intervention helped implementation; Support from HCPs and researchers facilitated adoption of approach | Ngangue et al., (2021); Ryan et al., (2022) |
|  | Delivery of support was well-designed | "*The commitment of the six weeks wasn’t major*" (Participant, O'Toole *et al.*, 2013) | HCPs perceived the intervention to be well designed and innovative; Participants enjoyed the components of the programme; Programme length was not seen as a big commitment; Structure of intervention valued by participants | Ngangue et al., (2021); Ryan et al., (2022); O’Toole et al., (2013) |
|  | Electronic tools useful for supporting self-management | "*Patient interviews indicated that they perceived the ePRO tool as a means to manage their conditions and health behaviors more holistically*" (Author quote; Khan *et al.*, 2018) | Electronic tool could support self-management in team environment; Electronic tool useful component in supporting self-management for multimorbidity; Patients felt the tool could help manage their conditions holistically | Khan et al., (2018); |
|  | Factors improving interdisciplinary collaboration and support | "*accessibility to relocated HCPs was well appreciated by patients*" (Author quote; Ngangue *et al.*, 2020) | Proximity between HCPs within clinics facilitated communication; Relocating HCPs to the same clinic improved access to these services for patients; Use of technology to conduct conference calls was a facilitator to interdisciplinary communication | Ngangue et al., (2020); Ngangue et al., (2021) |
|  | Feedback and monitoring from providers important to facilitate support | "*access to patient-reported data, that is, mood, health behaviors, etc, had helped them to explore trends in self-regulation and goal attainment over time*" (Author quote; Khan *et al.*, 2018) | Feedback from HCPs valuable to achieve goals; HCPs believed tool could be used for monitoring instead of appointments; HCPs could easily monitor self-regulation and goals; HCPs felt the tool could be used in remotely monitoring patient progress; Monitoring and advice from nurses led to medication adjustments; Monitoring by nurses allowed for early interventions; Patients felt tool should supplement in person appointments through regular feedback | Khan et al. (2018); Doyle et al. (2022) |
|  | Importance of follow up to check progress | "*I’d love if a morning was on where everybody could meet up to see how they were getting on instead of it just ﬁnishing*" (Patient; O'Toole *et al.*, 2013) | Caregiver was concerned about lack of follow up during intervention; Difficult to monitor patients' progress towards goals in the tool; HCPs able to follow up and monitor progress using support digital tool; HCPs emphasised the need for follow-up appointments; HCPs ensured follow up with patients; Issues with monitoring available for HCPs within electronic tool; Lack availability of HCPs caused problems for follow up; Nurses helped identify problems early; Nurses were responsive and noticed issues quickly; Patients appreciated the consistent follow up from nurses; Suggested that a follow up session could be added to group programme | Ngangue et al., (2020); Khan et al., (2018); O’Toole et al., (2013); Doyle et al. (2022) |
|  | Previous experience with providing support using these approaches | *This approach [motivational] was used in all other settings where I worked […]. So, we have already used it for several years*’ (Health care professional; Ngangue *et al.*, 2021)  “*I believe in it, and that is where we have to go more and more. It is aligned with the vision of our organization, but also the concept of chronic disease management”*  (Healthcare professional, Ngangue *et al*., 2021) | Familiar and comfortable with interdisciplinary collaboration; HCPs believe in the intervention principles; HCPs believed in the interdisciplinary approach to intervention; HCPs were familiar with intervention principles; Intervention approaches aligned with HCPs own values and visions; Motivational approach of the intervention was familiar to HCPs; Previous experience with intervention components facilitated implementation; Principles of intervention aligned with the vision and values of the health organisation | Ngangue et al., (2021) |
|  | Provision of support resources was beneficial | *I thought the book from beginning to end had a nice feeling about it, you didn’t feel “Oh God I can’t read this”. It’s great to have it, if something comes up you can look through it*" (Patient; O'Toole *et al.*, 2013) | Able to refer back to self-management resources provided when needed; Participant found the programme booklet clear and helpful; Participants benefitted from referrals to community resources; Positive impacts on wellbeing after referrals to other services; Self-management resources (e.g. CD) were beneficial; Self-management resources were beneficial | O’Toole et al., (2013); McCallum et al., (2021) |
|  | Recommendations for improving support delivery | *“I’m sorry it’s over, it would be nice if there were one or two weeks added and then a break"* (Participants; O'Toole *et al.*, 2013) | Involve more group activities as part of intervention; Need for linkage between the intervention tool and existing apps and services; Need for linking electronic tool with existing medical records; Participants wanted more feedback and encouragement from HCPs; Participants would like the programme to be extended; Patients desired for more engagement and support from health providers; Patients expected a more active role of providers; Patients suggested features including linking to other mHealth apps to expand tools scope; Recommendations to improve support for self-regulation; Recommended additional sessions targeting specific conditions; Require appointments at health centres for follow up; Smaller groups are more beneficial for participants; Suggested direct encouragement and acknowledgement from health providers via the tool | O’Toole et al., (2013); Khan et al., (2018); Ryan et al., (2022): Skou et al. (2023) |
|  | Sharing patient data between providers facilitated support and care | ‘*It is very facilitating; we see the notes of the doctors, the nutritionist, the nurses. Moreover, we know why we see him"* (Health care professional; Ngangue *et al.*, 2021) | Able to view data across multiple conditions and symptoms to make care decisions; Access to data allowed monitoring of progress; Easier access to patient reported data allowed identification of challenges facing patients; Nurses intervened with patient care when they noticed red flags in data; Programme allowed for patient data to be seen and shared; Sharing information via medical records supported implementation of intervention; Sharing medical records made consultations easier; Sharing patient notes facilitated the intervention | Doyle et al. (2022), Khan et al., (2018); Ngangue et al., (2020); Ngangue et al., (2021) |
|  | Support included education and learning strategies for self-management | *“I was forgetting my tablets. Now I set the table for the morning and put my tablets in the cup so I’ll see them and never forget them now" (*Patient; O'Toole *et al.*, 2013) | Educate on how to progress with goals; Educate participant on lifestyle behaviours; Education on strategies to manage symptoms; Goals related to self-management strategies (dietary management, smoking cessation); HCPs provide education to patients; HCPs provided participants with education and understanding of medication and symptoms; Improved understanding of medication; Participant learned fatigue management strategies from the intervention; Participants used mental health coping strategies; Use of medication management strategies to prevent forgetting to take medications | Doyle et al. (2022), O’Toole et al., (2013); McCallum et al., (2021) |
|  | Support providers changed current practice based on approaches used to deliver support | *So, when I do home visits now, I use the strengths based assessment*" (Health care professional; Fisher *et al.*, 2020) | Continued interdisciplinary support was important to sustain the programme; Intervention led to changes in practice for HCPs; Use of strength-based approach led to changes in current practice | Ryan et al., (2022); Fisher et al., (2020) |
|  | Support setting is important | *"I think the environment is really important, that we’ve taken them out of a health centre, we’ve taken them out of a health, be it a hospital outpatients or in our case it’s a health centre, but they’re not coming because their sick y’know it’s, the emphasis is off that" (*Facilitator; Ryan *et al.*, 2022) | Appreciated the non-health care setting; Leisure centre setting accessible at any time | Ryan et al., (2022) |
|  | Training in how to deliver support was important | *Finally, to ensure that they are adequately trained"* (Practice manager; Ngangue *et al.*, 2021) | HCPs felt that more training would be beneficial; Need to ensure that facilitators are appropriately trained in features of intervention; Not all HCPs were able to be trained in approach for intervention; Training for HCPs important; Training provided at start of intervention facilitated implementation; Training provided introduced HCPs to the approach so that they can apply it in practice; Training staff caused delays in intervention | Ngangue et al., (2021); Doyle et al. (2022) |
| ***Actual and perceived positive outcomes*** | Educational support was useful | "*promote knowledge development and educate patients had benefits on patients’ self-management*." (Author quote; Ngangue *et al.*, 2020) | Educating participants led to improved self-management; Education was useful, but no major impact on daily life; Patients improved their understanding of their conditions | Ngangue et al., (2020); McCallum et al., (2021); Doyle et al. (2022) |
|  | Support led to less healthcare utilisation | “*You’re catching them [through ProACT], so if they go to their GP for an antibiotic, they’re not ending up in hospital and that’s huge because it’s a win-win for everybody*”  (Nurse; Doyle *et al.*, 2022) | Fewer hospital admissions and healthcare utilisation as a result of the support programme | Doyle et al. (2022) |
|  | Moderate improvement in health | "*three described slight improvement that did not impact daily life*" (Author quote; McCallum *et al.*, 2021) | Slight improvement that did not impact daily life | McCallum et al., (2021) |
|  | Support led to improvements in behavioural outcomes | ‘*so, it’s great, their, their conﬁdence is up even just to go and do that… you know it’s brilliant, even to see that people are coming once the programme’s ﬁnished you know’* (Facilitator; Ryan *et al.*, 2022) | Electronic tool helped achieve short-term outcomes related to successful self-management; Improved confidence and self-esteem of patients; Improved self-management behaviours; Improvements in motivation; Increased self-efficacy and self-management behaviours; Intervention improved the confidence of participants; Motivational approach of intervention improved management of conditions; Participants noted improvements in confidence due to intervention; Self-management support approach improved confidence and self-esteem; Support provided confidence to patients | Doyle et al. (2022), Khan et al., (2018); Ngangue et al., (2020); McCallum et al., (2021); Fortin et al., (2021a); Ryan et al., (2022) |
|  | Support led to improvements in health outcomes | *“There are many patients who have taken care of themselves and who have stopped certain medications, who have managed to control their diabetes or even their cholesterol without medication”*, (Health care professional; Fortin *et al.*, 2021a)  *‘I don’t feel as tired as I used to, y’know…. I feel I got more energy, y’know I feel because of my problem y’know with my leg as well I feel that my muscles, my body is stronger*’  (Patient, Ryan *et al.,* 2022) | Family member reported weight loss and improvement in patient's health; Family members and patients perceived improvements in quality of life; HCPs observed improvements in patient health status; Improved energy levels and physical strength; Improvements in quality of life; Improvements in range of health outcomes; Patients reported positive clinical outcomes; Physical improvements including strength and movement | Fortin et al., (2021a); Ryan et al., (2022); Doyle et al. (2022) |
|  | Support led to improvements in lifestyle behaviours | “*I would skip a meal now and again, I’m more conscious now that I need to eat four times a day*" (Participant, O'Toole *et al.*, 2013) | Improvements in diet and monitoring health; Improvements in nutrition and physical activity; Lifestyle changes were reported; More conscious regarding dietary habits | Fortin et al., (2021a); O’Toole et al., (2013) |
|  | Support led to improvements in mental health outcomes | *Both the facilitators (physiotherapists and ﬁtness instructors) and study participants identiﬁed signiﬁcant beneﬁts to the Activ8 programme both physically and psychologically.* (Author quote; Ryan *et al.*, 2022) | Improvement in wellbeing impacted daily life; Physical and psychological benefits of intervention | McCallum et al., (2021); Ryan et al., (2022) |
|  | Support led to positive perceptions of their health | "*I expect to live longer than I would have believed a few months ago.* " (Patient; Ngangue *et al.*, 2020) | Change perceptions of their conditions and life; Increased focus on their own health; Open minded to trying things that may help | Ngangue et al., (2020); McCallum et al., (2021) |
|  | Support motivated people to engage with health care services | "*she had attended a local mental health group (after signposting from her GP) and had referred herself to a counselling service*." (Author quote; McCallum *et al.*, 2021) | Attendance at signposted and referred services; Consultations motivated participants to seek help for issues; Improved attendance at health visits; Patients engaged with advice provided by HCPs; Patients visited GPs due to the support and advice received; Signpost to health services and resources | McCallum et al., (2021); Doyle et al. (2022) |
|  | Support received improved engagement in social and daily activities | “*I’m going out a bit more, just out for an hour here and there*" (Patient; O'Toole *et al.*, 2013) | Engagement in social and daily activities improved wellbeing; Greater engagement in daily activities; Group intervention improved engagement in domestic activities; Group intervention increased community participation; Group intervention increased confidence for social activities; Group intervention increased motivation for engaging in activities; Group intervention increased participation in exercise classes; Increased engagement in social activities; Increased participation in social activities; Motivation to increase social activities; Motivation to re-engage in social activities; Self-management resources provided motivation to re-engage in social activities | McCallum et al., (2021); O’Toole et al., (2013); Ryan et al., (2022) |
|  | Support was beneficial to those who received it | *Most of the participants felt the CARE Plus intervention was beneﬁcial and valuable in some way, even if it did not result in major improvement in daily life*" (Author quote; McCallum *et al.*, 2021) | Intervention perceived as beneficial and valuable; No issues with motivation to do sessions at home; Self-management support sessions were useful and relevant; Support from nurses alongside digital platform was beneficial | McCallum et al., (2021); Skou et al. (2023); Doyle et al. (2022) |

**S6 Table. Preferred Reporting Items for Systematic Reviews and Meta-Analyses (PRISMA) Checklist 2020.** Completed PRISMA checklist for reporting systematic reviews, with page references and text excerpted from the manuscript.

| **Section and Topic** | **Item #** | **Checklist item** | **Location where item is reported** |
| --- | --- | --- | --- |
| **TITLE** | | |  |
| Title | 1 | Identify the report as a systematic review. | Title page – “Effectiveness and experiences of self-management interventions in adults with multiple long-term conditions: a mixed methods systematic review” |
| **ABSTRACT** | | |  |
| Abstract | 2 | See the PRISMA 2020 for Abstracts checklist. | See PRISMA abstract checklist |
| **INTRODUCTION** | | |  |
| Rationale | 3 | Describe the rationale for the review in the context of existing knowledge. | Page 5 |
| Objectives | 4 | Provide an explicit statement of the objective(s) or question(s) the review addresses. | Page 5 |
| **METHODS** | | |  |
| Eligibility criteria | 5 | Specify the inclusion and exclusion criteria for the review and how studies were grouped for the syntheses. | Page 6 and 7 – under subheading “Eligibility criteria” |
| Information sources | 6 | Specify all databases, registers, websites, organisations, reference lists and other sources searched or consulted to identify studies. Specify the date when each source was last searched or consulted. | Page 7 – under subheading “Information sources and search strategy” |
| Search strategy | 7 | Present the full search strategies for all databases, registers and websites, including any filters and limits used. | Page 7 and 8 – under subheading “Information sources and search strategy” and in S1 Fig |
| Selection process | 8 | Specify the methods used to decide whether a study met the inclusion criteria of the review, including how many reviewers screened each record and each report retrieved, whether they worked independently, and if applicable, details of automation tools used in the process. | Page 8– under subheading “Study selection” |
| Data collection process | 9 | Specify the methods used to collect data from reports, including how many reviewers collected data from each report, whether they worked independently, any processes for obtaining or confirming data from study investigators, and if applicable, details of automation tools used in the process. | Page 8 to 10 – under subheadings “Data Extraction” and “Data synthesis” |
| Data items | 10a | List and define all outcomes for which data were sought. Specify whether all results that were compatible with each outcome domain in each study were sought (e.g. for all measures, time points, analyses), and if not, the methods used to decide which results to collect. | Page 6 – under subheading “Eligibility Criteria” |
|  | 10b | List and define all other variables for which data were sought (e.g. participant and intervention characteristics, funding sources). Describe any assumptions made about any missing or unclear information. | Page 8 – under subheading “Data extraction” |
| Study risk of bias assessment | 11 | Specify the methods used to assess risk of bias in the included studies, including details of the tool(s) used, how many reviewers assessed each study and whether they worked independently, and if applicable, details of automation tools used in the process. | Page 8 to 9 – under subheading “Quality Appraisal” |
| Effect measures | 12 | Specify for each outcome the effect measure(s) (e.g. risk ratio, mean difference) used in the synthesis or presentation of results. | Page 9 and 10 – under subheading “Data synthesis” |
| Synthesis methods | 13a | Describe the processes used to decide which studies were eligible for each synthesis (e.g. tabulating the study intervention characteristics and comparing against the planned groups for each synthesis (item #5)). | Page 9 and 10 – under subheading “Data synthesis” |
|  | 13b | Describe any methods required to prepare the data for presentation or synthesis, such as handling of missing summary statistics, or data conversions. | N/A |
|  | 13c | Describe any methods used to tabulate or visually display results of individual studies and syntheses. | Page 9 – under subheading “Data synthesis” |
|  | 13d | Describe any methods used to synthesize results and provide a rationale for the choice(s). If meta-analysis was performed, describe the model(s), method(s) to identify the presence and extent of statistical heterogeneity, and software package(s) used. | Page 9 and 10 – under subheading “Data synthesis” |
|  | 13e | Describe any methods used to explore possible causes of heterogeneity among study results (e.g. subgroup analysis, meta-regression). | Page 9 and 10 – under subheading “Data synthesis” |
|  | 13f | Describe any sensitivity analyses conducted to assess robustness of the synthesized results. | N/A |
| Reporting bias assessment | 14 | Describe any methods used to assess risk of bias due to missing results in a synthesis (arising from reporting biases). | N/A |
| Certainty assessment | 15 | Describe any methods used to assess certainty (or confidence) in the body of evidence for an outcome. | N/A |
| **RESULTS** | | |  |
| Study selection | 16a | Describe the results of the search and selection process, from the number of records identified in the search to the number of studies included in the review, ideally using a flow diagram. | Page 11 – under “Results”  Figure 1 – PRISMA Flow Diagram |
|  | 16b | Cite studies that might appear to meet the inclusion criteria, but which were excluded, and explain why they were excluded. | N/A |
| Study characteristics | 17 | Cite each included study and present its characteristics. | Table 1, 2 and 3 – study characteristics |
| Risk of bias in studies | 18 | Present assessments of risk of bias for each included study. | Page 13 – under subheading ‘Quality Appraisal’  Tables 5 to 9 - quality appraisal tables and results |
| Results of individual studies | 19 | For all outcomes, present, for each study: (a) summary statistics for each group (where appropriate) and (b) an effect estimate and its precision (e.g. confidence/credible interval), ideally using structured tables or plots. | Page 13 to 16 – under subheading “Quantitative synthesis” |
| Results of syntheses | 20a | For each synthesis, briefly summarise the characteristics and risk of bias among contributing studies. | Page 13 to 19 – under subheading “Quantitative synthesis” and “Qualitative synthesis” |
|  | 20b | Present results of all statistical syntheses conducted. If meta-analysis was done, present for each the summary estimate and its precision (e.g. confidence/credible interval) and measures of statistical heterogeneity. If comparing groups, describe the direction of the effect. | Page 13 to 16 – under subheading “Quantitative synthesis” |
|  | 20c | Present results of all investigations of possible causes of heterogeneity among study results. | Page 13 to 16 – under subheading “Quantitative synthesis” |
|  | 20d | Present results of all sensitivity analyses conducted to assess the robustness of the synthesized results. | N/A |
| Reporting biases | 21 | Present assessments of risk of bias due to missing results (arising from reporting biases) for each synthesis assessed. | N/A |
| Certainty of evidence | 22 | Present assessments of certainty (or confidence) in the body of evidence for each outcome assessed. | N/A |
| **DISCUSSION** | | |  |
| Discussion | 23a | Provide a general interpretation of the results in the context of other evidence. | Page 21 to 23– under subheading “Comparison with previous research” |
|  | 23b | Discuss any limitations of the evidence included in the review. | Page 23 to 24 – under subheading “Strengths and Limitations”– |
|  | 23c | Discuss any limitations of the review processes used. | Page 23 to 24 – under subheading “Strengths and Limitations”– |
|  | 23d | Discuss implications of the results for practice, policy, and future research. | Page 24 and 25 – under subheading “Implications for future research” and “Implications for future practice” |
| **OTHER INFORMATION** | | |  |
| Registration and protocol | 24a | Provide registration information for the review, including register name and registration number, or state that the review was not registered. | PROSPERO - CRD42022302959 (Jan 2022) |
|  | 24b | Indicate where the review protocol can be accessed, or state that a protocol was not prepared. | <https://www.crd.york.ac.uk/prospero/display_record.php?RecordID=302959> PROSPERO (CRD42022302959) |
|  | 24c | Describe and explain any amendments to information provided at registration or in the protocol. | N/A |
| Support | 25 | Describe sources of financial or non-financial support for the review, and the role of the funders or sponsors in the review. | This study is supported by the National Institute for Health and Care Research (NIHR) Applied Research Collaboration East Midlands (ARC EM). The views expressed in this publication are those of the authors and not necessarily those of the National Institute for Health and Care Research or the Department of Health and Social Care.  The funders had no role in study design, data collection and analysis, decision to publish, or preparation of the manuscript. |
| Competing interests | 26 | Declare any competing interests of review authors. | The author(s) declared no potential conflicts of interest with respect to the research, authorship, and/or publication of this article |
| Availability of data, code and other materials | 27 | Report which of the following are publicly available and where they can be found: template data collection forms; data extracted from included studies; data used for all analyses; analytic code; any other materials used in the review. | Data are publicly available |

*From:* Page MJ, McKenzie JE, Bossuyt PM, Boutron I, Hoffmann TC, Mulrow CD, et al. The PRISMA 2020 statement: an updated guideline for reporting systematic reviews. BMJ 2021;372:n71. doi: 10.1136/bmj.n71. This work is licensed under CC BY 4.0. To view a copy of this license, visit <https://creativecommons.org/licenses/by/4.0/>
